# Ethnopharmacology of *Pinus* species with focus on the Hispaniola pine (*Pinus occidentalis* Swartz): evidence, gaps, and research roadmap

**DOI:** 10.3389/fphar.2025.1680390

**Published:** 2025-11-13

**Authors:** Alberto J. Núñez-Selles, Lauro Nuevas-Paz, Elisa A. Gómez-Torres

**Affiliations:** 1 Research Division, Universidad Nacional “Pedro Henríquez Ureña” (UNPHU), Santo Domingo, Dominican Republic; 2 Technology Research and Development Center (TECUNEV), Universidad Nacional Evangélica (UNEV), Santo Domingo, Dominican Republic; 3 Department of Pharmaceuticals and Bioprospecting, Instituto de Innovación en Biotecnología e Industria (IIBI), Santo Domingo, Dominican Republic

**Keywords:** *Pinus sp*, *Pinus occidentalis* Swartz, ethnopharmacology, pharmacology, phytochemical composition, polyphenols, proanthocyanidins, Hispaniola

## Abstract

The review systematically maps the ethnomedicinal uses and chemistry of *Pinus* spp., with an emphasis on the under-studied *Pinus occidentalis* Swartz, known as “pino criollo”, “pino de cuaba” or “pin creole”. It is the only native pine species of Hispaniola, and holds ecological, cultural, and medicinal significance across the Dominican Republic and Haiti. A data search was conducted across several databases (Google Scholar, SciFinder, Scopus, ScienceDirect, PubMed/Medline, and TRIP) to evaluate the existing knowledge of *P. occidentalis* spp. and compare it to that of other *Pinus* species for medicinal uses. The search showed evidence about the medicinal use of this pine species for treating respiratory ailments (cough, cold, and flu), skin infections, wounds, and inflammatory conditions, mainly through hot decoctions of pine needles, bark, cone tender sprouts, and the resin in some locations in Hispaniola. Still, phytochemical data were scarce, limited to the composition of the needle’s essential oil and resin’s turpentine oil in the 20th century. Systematic pharmacological validation of these limited ethnopharmacological findings is still pending, along with the determination of phytochemicals. Research on *P. occidentalis* shows potential as a natural health product. The urgent need for sustainable strategies is emphasized by conservation concerns related to habitat loss and deforestation. Future research should focus on detailed ethnopharmacology, conservation and propagation techniques for its exploitation, extraction technologies, chemical profiling, and pharmacological screening according to ethnomedicinal surveys, to set *P. occidentalis* as a promising candidate for phytotherapeutic development and integrative health applications in Hispaniola. These gaps underscore the need for a research roadmap of this endemic tree across the island. The review represents the first comprehensive synthesis of the ethnomedical applications of *P. occidentalis* Swartz, systematically mapping its cultural and therapeutic significance across Hispaniola.

## 1 Introduction

The genus *Pinus* (family *Pinaceae*) is one of the most widespread and economically significant groups of coniferous trees, comprising over 120 species distributed across various ecosystems, from boreal forests to temperate and subtropical regions (Critchfield and Little, 1966; Diekmann et al., 2002). *Pinus* spp. have played a crucial role in human history, serving as sources of timber, resin, and medicinal compounds. Their pharmacological properties have been extensively documented across different cultures, highlighting their use for treating a wide range of ailments (Bhardwaj et al., 2021; Dziedziński et al., 2021; Li et al., 2025). Pine extracts have transitioned from being overlooked as waste in the timber industry to being recognized as a powerful source of metabolites with significant health and medicinal benefits. These extracts offer a wide range of health benefits, including strong antioxidant, anti-inflammatory, and neuroprotective effects, often achieved through interactions with other compounds or the intestinal microflora (Sun and Shahrajabian, 2023; Sánchez-Moya et al., 2024; Song et al., 2024). Although substantial progress has been made regarding their applications in food and pharmaceuticals, the cellular and molecular mechanisms driving their efficacy remain largely unexplored. This gap is largely due to the variability in growth environments, extraction methods, and other factors.

Ethnopharmacology has provided substantial evidence regarding the therapeutic applications of *Pinus* spp. (Sharma et al., 2018; El Omari et al., 2021; Shah et al., 2024). Indigenous communities, herbalists, and healers in different parts of the world have utilized the stem bark, needles, resin, cones, and seeds of pine trees for treating inflammatory diseases, respiratory disorders, microbial infections, and even metabolic conditions such as diabetes. In Latin America, *Pinus* sp. holds a prominent place in folk medicine, particularly in Mexico (Mayo-Mayo et al., 2024). Similarly, North American indigenous groups have relied on pine extracts for their antibacterial and antioxidant properties (Royer et al., 2013; Uprety et al., 2013; Lans, 2016). Recent phytochemical and pharmacological research has confirmed that *Pinus* spp. contain bioactive metabolites, such as flavonoids, tannins, terpenes, and proanthocyanidins, which contribute to their medicinal properties (Chammam et al., 2024; Sharma et al., 2025). These metabolites exhibit antioxidant, anti-inflammatory, antimicrobial, and neuroprotective effects, making *Pinus* an important natural source for potential therapeutic applications.

*Pinus occidentalis* Swartz, commonly known as Hispaniola pine or “pino criollo”, or “pino de cuaba” or “pin creole”, is an endemic conifer species found in the mountainous regions of the Dominican Republic and Haiti (Speer et al., 2004). It is the only native pine species in Hispaniola and plays a crucial ecological role in maintaining biodiversity and stabilizing soil in high-altitude forests (Chardon, 1941). The first published report on the description of *P. occidentalis* in the island was made by Swartz in 1788 (Urban, 1902). However, it was not until the end of the 1990s that the first report on the taxonomy and ecology of this species was published (Darrow and Zanoni, 1990). According to the classification published by Critchfield and Little (1966), *P. occidentalis* Swartz belongs to the genus *Pinus*, section *Pinus*, a group of coniferous trees characterized by having two fibrovascular bundles in their needles, which are generally hard and woody, and open at maturity, comprising 62 species. In its subsection (*Australes*), *P. occidentalis* is found along with 10 other species of pines (*palustris* Mill.*, taeda* L.*, echinata* Mill.*, glabra* Walter*, rigida* Mill.*, serotina* Michx.*, pungens* Lamb.*, elliottii* Engelm*, caribaea* Morelet, and *cubensis* Griseb). *P. occidentalis* Swartz can be found at altitudes between 200 and 3,200 m above sea level (Farjon and Styles, 1997), and up to 41 halotypes with high genetic diversity have been found (Rodriguez de Francisco et al., 2022). The seeds are small with long detachable wings, and the leaves are 10–15 cm in length. Each tree has both female and male flowers, and the cones measure 6–8 cm in length, bearing winged seeds (Bueno-Lopez, 2009). However, reports about its medicinal uses and the phytochemical composition of the tree parts are scarce. This review examines the ethnopharmacological knowledge of *Pinus* spp. from the same subgenus and section across various regions, analyzing historical records, ethnobotanical studies, and recent pharmacological findings to inform future research on *P. occidentalis* as a potential source of nutritional and medicinal products.

## 2 Data search

**FIGURE 1:**
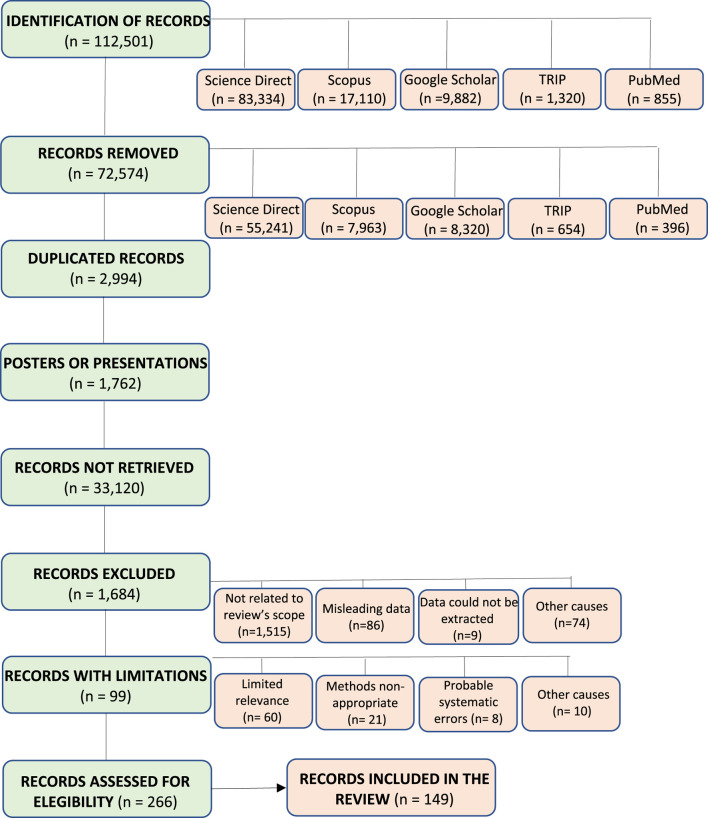
Flow chart diagram for the selection of records from databases.

An extensive search was conducted using published reports from specialized data sources without date constraints, including Google Scholar, SciFinder, Scopus, ScienceDirect, PubMed/Medline, and the TRIP Database. The initial keyword setting (*Pinus* spp. and ethnopharmacology) yielded 112,501 records, which were further refined by removing those not directly related to the subgenus *Pinus*. Duplicated records and records from poster or oral presentations at scientific meetings were also removed. A second keyword setting (chemical composition and pharmacological evaluation) rendered 358 records, and a third and final refinement included *P. occidentalis* Swartz. The search included studies published in the XX and XXI centuries. After refining the data across all databases, 266 records were registered. These records included species of the same subsection (*Australes*) where *P. occidentalis* is included, and some species from another section (*Ternatae*) for comparison purposes. Out of these, 149 were included in the review as shown in Figure 1.

## 3 *Pinus* spp. in ethnopharmacology (subgenus *Pinus*)

Intensive research on the ethnopharmacology of *Pinus* spp. has primarily focused on a few specific varieties, with *P. pinaster* being the most extensively studied. The bark of *P*. *pinaster* has attracted significant attention due to its medicinal properties, which led to the development of Pycnogenol^®^, a standardized extract derived from its bark (Ferreira-Santos et al., 2020). Additionally, both *P*. *sylvestris* and *P*. *roxburghii* have been studied, and their findings are discussed in the following sections. Other species with limited bibliographic records have been grouped in a separate section.

### 3.1 Pinus pinaster Aiton

The maritime pine (*P. pinaster* Alton) is a coniferous tree native to the Mediterranean region, particularly found in coastal areas of France, Spain, Portugal, and North Africa, belonging to the same section, subgenus, and section (*Pinus*) as *P. occidentalis* (Bouffier et al., 2013). Among the various parts of *P. pinaster*, including its needles, resin, and seeds, the bark has garnered the most attention in ethnopharmacology due to its wealthy phytochemical profile (Dridi and Bordenave, 2020; Alonso-Esteban et al., 2022). The pine bark has been used in folk medicine for wound healing, respiratory ailments, and circulatory disorders (Maimoona et al., 2011).

Pycnogenol^®^, the standardized extract from *P. pinaster* bark, was first developed in the 1960s by Charles Haimoff in Berlin; however, its chemical composition was not elucidated until the 1980s (Rohdewald, 2002). The extract was later commercialized by Horphag Research, which secured exclusive global rights and began distributing Pycnogenol^®^ as a branded nutraceutical. Its formal introduction into the market occurred in the early 1970s, and it has since expanded to over 80 countries, supported by more than 450 scientific publications validating its antioxidant, anti-inflammatory, and vascular benefits (Pycnogenol, 2025). This product is rich in procyanidins, flavonoids, and phenolic acids, which contribute to its antioxidant, anti-inflammatory, and cardiovascular benefits (Rohdewald, 2002). Modern research has validated the therapeutic potential of Pycnogenol^*®*^, demonstrating its efficacy in improving blood circulation, reducing oxidative stress, and supporting cognitive function (Weichmann and Rohdewald, 2024). Studies have shown that it enhances vascular health by promoting nitric oxide production, leading to improved endothelial function and a reduction in hypertension (Zhang et al., 2019). The bark extract has also shown promise in managing diabetes by improving blood sugar regulation and reducing complications associated with oxidative damage (Mohammadi et al., 2025). Its bioavailability and metabolism have been studied, confirming its rapid absorption and distribution in various tissues, including blood cells and synovial fluid (Bayer and Högger, 2024). The broad spectrum of benefits associated with *P. pinaster* bark extract underscores its significance in both traditional and modern medicine, making it a valuable natural remedy with extensive clinical applications (Maimoona et al., 2011).

*P. pinaster* bark contains high concentrations of polyphenols, flavonoids, and procyanidins, which contribute to its antioxidant, anti-inflammatory, and cardiovascular benefits (Iravani and Zolfaghari, 2014; Alonso-Esteban et al., 2022). The content of polyphenols in the pine bark could be 1.5 times higher than in the other parts of the tree (Sadeghi et al., 2014). These metabolites help reduce oxidative stress and enhance vascular health. *P. pinaster* bark has been used for treating respiratory and circulatory disorders, as well as for wound healing, with historical records indicating its use in Turkey herbal remedies (Tümen et al., 2018). The bark’s vasorelaxant properties make it particularly valuable for improving blood flow and reducing hypertension, a benefit that has been studied in modern pharmacology (Mohammadi et al., 2025). Unlike the needles or resin, which primarily serve for antimicrobial and skin treatment purposes, respectively, the bark’s complex polyphenolic composition provides a broader spectrum of therapeutic effects, making the bark the most attractive part of the tree for medicinal applications.

### 3.2 Pinus sylvestris L.

*P. sylvestris* L., another member of the *Pinus* section, is one of the most widely distributed pine species, spanning across Europe and Asia, and has been studied for its medicinal properties (Brichta et al., 2023). Various parts of the tree, including its bark, needles, and resin, have been used in herbal medicine for treating respiratory infections, skin conditions, and inflammatory disorders (Laavola et al., 2015). Among these, the bark and needle extracts have received significant attention due to their rich phytochemical composition, including a diverse array of metabolites, like polyphenols, flavonoids, tannins, terpenes, and lignans, which contribute to their pharmacological effects (Kim et al., 2021). The bark and needle extracts are rich in polyphenolic compounds such as catechin, epicatechin, and quercetin derivatives (Metsämuuronen and Sirén, 2019). These metabolites exhibit strong antioxidant and anti-inflammatory properties, making *P. sylvestris* valuable in managing oxidative stress-related conditions (Karonen et al., 2004; Qinfeng et al., 2020). It also contains condensed tannins, which contribute to its antimicrobial effects (Nisca et al., 2021; Mirković et al., 2024). This class of metabolites has several pharmacological effects, depending on the type of tannins used, such as antimicrobial, antioxidant, antiviral, and restoration of the intestinal microbiota (de Melo et al., 2023).

The needles of *P. sylvestris* are rich in monoterpenes such as α-pinene, β-pinene, limonene, and camphene, which provide antimicrobial, expectorant, and bronchodilator effects (Ioannou et al., 2014). These metabolites are particularly beneficial for respiratory health, explaining the traditional use of Scots pine needle infusions for colds and bronchitis (Kazantsev et al., 2022). The bark of *P. sylvestris* contains lignans, including pinoresinol and matairesinol, which have shown neuroprotective and anti-cancer properties in pharmacological studies. Additionally, it contains phenolic acids such as gallic, ferulic, and caffeic acids, which contribute to the anti-inflammatory and cardiovascular benefits of *P. sylvestris* extracts. (Metsämuuronen and Sirén, 2019). The pharmacological relevance of *P. sylvestris* in cancer chemotherapy has been substantiated through *in vitro* studies demonstrating its selective cytotoxicity and pro-apoptotic effects against various human cancer cell lines (Hoai et al., 2015). Pine needle extract showed lower IC50 values in receptor-negative breast cancer cells (MDA-MB-231) than in receptor-positive cells (MCF-7). Moreover, the bark extract was cytotoxic against HeLa cells, inducing over 70% of apoptosis at 200 μg/mL.

The presence of phytostilbenes in polyphenolic extracts of *P. silvestris* and other members of the *Pinus* spp., such as pinosylvin (3,5-dihydroxy-*trans*-stilbene), has been explored for anticancer treatment by modulating MAPK, ERK, and PI3K pathways (Bakrim et al., 2022; Tshikhudo et al., 2025). The effects of pinosylvin (0–80 μM) on the metastatic capacities of SAS, SCC-9, and NSC-3 oral cancer cells have been evaluated (Chen et al., 2021). Pinosylvin suppressed matrix metalloproteinase 2 (MMP-2) but enhanced the expression of tissue inhibitors of MMP-2. It has also been tested on nasopharyngeal carcinoma cells, and a dose-effect relationship could be observed on NPCO39 and NPCBM line cells (Chuang et al., 2021). Pinosylvin has also inhibited colorectal cancer cells (HCT-116) proliferation (Park et al., 2013). This class of metabolites has been gaining the attention of the scientific community as possible candidates for cancer therapies.

### 3.3 Pinus roxburghii Sarg.

*P. roxburghii* Sarg., commonly known as chir pine, is a dominant conifer species in the Himalayan region, particularly in India, Nepal, and Bhutan (Chowdhary et al., 2025). It has been utilized by native populations for its therapeutic properties, with various parts of the tree (bark, needles, resin, and knotwood) being employed for treating inflammatory conditions, respiratory ailments, and microbial infections (Singh et al., 2019). Among these, the oleoresin and bark extracts have received significant attention due to their rich phytochemical composition and pharmacological potential (Aman et al., 2023). The oleoresin of *P. roxburghii* is particularly rich in monoterpenoids and sesquiterpenoids, including α-pinene, β-pinene, Δ^3^-carene, and limonene, which exhibit antioxidant (Tsvetkov et al., 2019) and antimicrobial (Ayub et al, 2025) effects. Healers have also employed *P. roxburghii* oleoresin for wound healing and pain relief (Kaushik et al., 2013). A hot decoction of the leaves is applied locally to treat sprains. The resin is applied to boils, heel cracks, and on either side of the head just above the eye to alleviate swelling. Persons suffering from tuberculosis were advised to stay for 2 months in a hut built in the pine forest to aid in recovery from the disease (Singh et al., 1990). Leaves and resin have also been used for treating measles and warts, respectively (Mehmood Abbasi et al., 2010).

The bark and knotwood extracts contain flavonoids such as quercetin, kaempferol, and catechins, which contribute to its antioxidant properties (Mehmood et al., 2024). Pinoresinol and matairesinol in these extracts have demonstrated cardiovascular benefits (Milder et al., 2005), including also gallic, ferulic, and caffeic acids, which enhance the anti-inflammatory and antimicrobial potential of *P. roxburghii* bark extracts (Mehmood et al., 2024). As compared to other pine species grown in India (*P. gerardiana* Wall. and *P. wallichiana* A.B. Jacks.), the hydroalcoholic bark extract of *P. roxburghii* has shown higher anti-inflammatory effects (Sharma et al., 2016).

### 3.4 Other *Pinus* sp.

The ethnopharmacological knowledge of other *Pinus* spp. underscores their significance in traditional medicine across different regions. For example, the Himalayan species as *P. wallichiana* A.B. Jacks, is known for its medicinal applications in Tibetan and Ayurvedic medicine (Sinha, 2019). Medicinal uses include various parts of the plant or resin for curing various ailments, such as healing, fever, and bacterial diseases (Sinha, 2019). The essential oils (EOs) extracted from the needles have demonstrated antimicrobial and antioxidant properties, making them valuable in herbal formulations (Nazish et al., 2018). In Brazil, *P. elliottii* Engelm, known as slash pine, is valued for its resin, which is applied topically for skin conditions and burns (de Sousa et al., 2024). Studies have explored the phytochemical composition of its essential oil, highlighting its potential for pharmacological applications (Pagula and Baeckström, 2006). North American indigenous groups, including the Cherokee and Navajo tribes, have used *P. ponderosa* Douglas bark infusions for respiratory ailments (Erichsen-Brown, 1989). The needles are brewed into teas to boost immunity and alleviate cold symptoms. The EO of this *Pinus* sp. has shown a remarkable antifungal activity *in vitro* (Baranowska et al., 2002). While the *P. ponderosa* EO had a 100% growth inhibition of *Fusarium colmorum* and *Fusarium solani,* the *P. resinosa* Sol, known as red pine, and the *P. strobus* L. known as white pine, EOs showed inhibition of growth below 90%.

The Algonquin people have traditionally used *P. strobus* needle tea as an immune booster (Uprety et al., 2013). The bark and the resin have been used to treat infections and inflammatory conditions. Modern research has confirmed its antioxidant properties (Oshetkova and Klimowicz, 2025). It has been reported in the Balkan region that the needle’s EO from three pine species (*P. heldreichii* Christ, *P. peuce* Griseb, and *P. mugo* Turra) exhibits anti-inflammatory and potential anti-cancer effects (Basholli-Salihu et al., 2017). An ethnobotanical study in Turkey about medicinal uses of *P. brutia* Ten., *P. nigra* J.F. Arnold, *P. pinea* L., and *P. sylvestris* L. showed that *P. nigra* was the most preferred and used *Pinus* sp. for medicinal purposes (Kızılarslan and Sevgi, 2013).

Ethnomedicinal studies on *P. halepensis* Mill. have demonstrated its use as a protective remedy against respiratory and digestive disorders, arterial hypertension, and microbial infections. These medicinal uses vary based on the part used and the region (El Omari et al., 2021). Its extracts and EOs have demonstrated several biological effects, including antimicrobial, antidiabetic, anti-inflammatory, cytotoxic, antiparasitic, and hepatoprotective properties.

## 4 Pinus occidentalis Swartz

**FIGURE 2:**
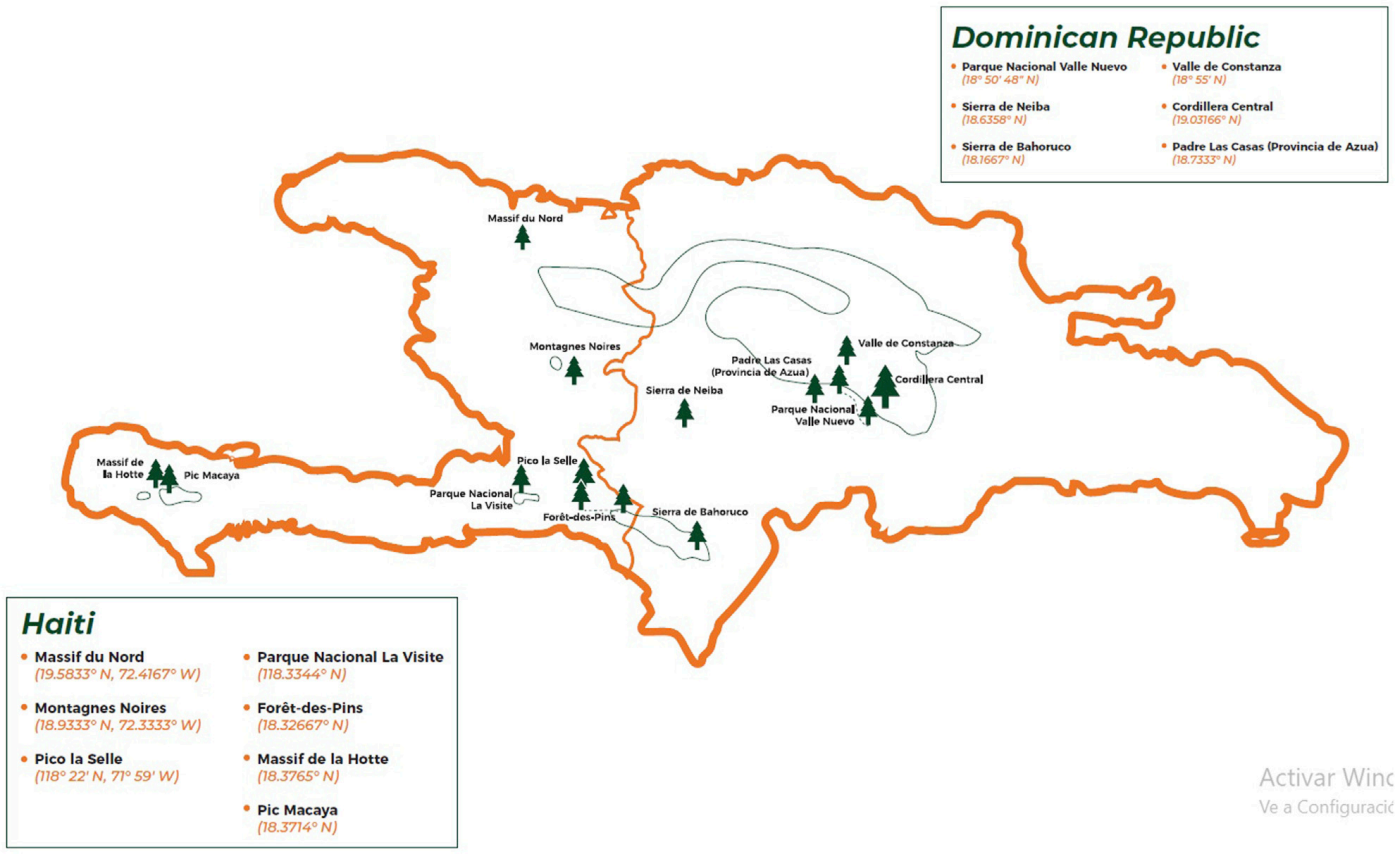
The distribution range of *Pinus occidentalis* Swartz in Hispaniola (Source: Instituto de Innovación en Biotecnología e Industria -IIBI-, Santo Domingo, Dominican Republic).

*P. occidentalis* Swartz is endemic to the Dominican Republic and Haiti. It is known as “pino criollo” or “pino de cuaba” (Dominican Republic) or “pin créole” (Haiti) (see Figure 2). It is the only native pine species in Hispaniola, with an estimated area of 3,500 km^2^ in 1995 for the Dominican Republic (Bueno-Lopez et al., 2024), but there is no precise data about Haiti. The taxonomy, ecology, and history of exploitation up to the 20th century have been published in a single and useful report (Darrow and Zanoni, 1990). It is the main timber species in Hispaniola, comprising approximately 95% of all timber harvested. Despite its economic importance, estimating inventory levels and accounting for harvested volume is difficult because no standardized system is used for volume appraisal and inventory purposes. There are more than 400 records on the biology and cultivation, propagation, and timber uses of *P. occidentalis* on Hispaniola (BVEARMB, 2025).

The Hispaniola pine seeding has been studied in Haiti for reforestation and soil restoration (Hubbel et al., 2018). Its proteome with seeds and pollen samples has been characterized in the Dominican Republic, indicating a high content of storage protein, stress response, and metabolism-related proteins in both the seed and the pollen (Rodriguez de Francisco et al., 2016). The variation of wood density in forests of a specific region (La Sierra, Dominican Republic) has been studied, indicating the influence of the species (as compared to the Caribbean pine), the tree age, and relative height on wood production (Bueno-Lopez et al., 2025). Research on its genetic diversity and adaptability to environmental stressors for optimizing propagation techniques and assessing its resilience to climate change has been reported (Perez Santana et al., 2008). The main use of *P. occidentalis* in this region is as firewood or wood for the construction of houses.

It is one of the plants most used as a hot drink (bark) for cough, cold, and flu in the Central Mountains of the Dominican Republic, especially in the area of the “*Juan P. Rancier*” National Park, Valle Nuevo (Peguero, 2002). The bark from the pine stem is put in boiling water for 2–3 h and filtered. The dosage and frequency of administration of this hot drink are determined by traditions passed down orally. However, the most used part in this region is the tender shoots of pine cones, especially in the highest parts, and needles for use as a tonic in the treatment of flu, prepared in the same way as the bark. Additionally, its resin has been applied topically for wound healing and as an antiseptic (Schulz Calvo, 2014).

**FIGURE 3:**
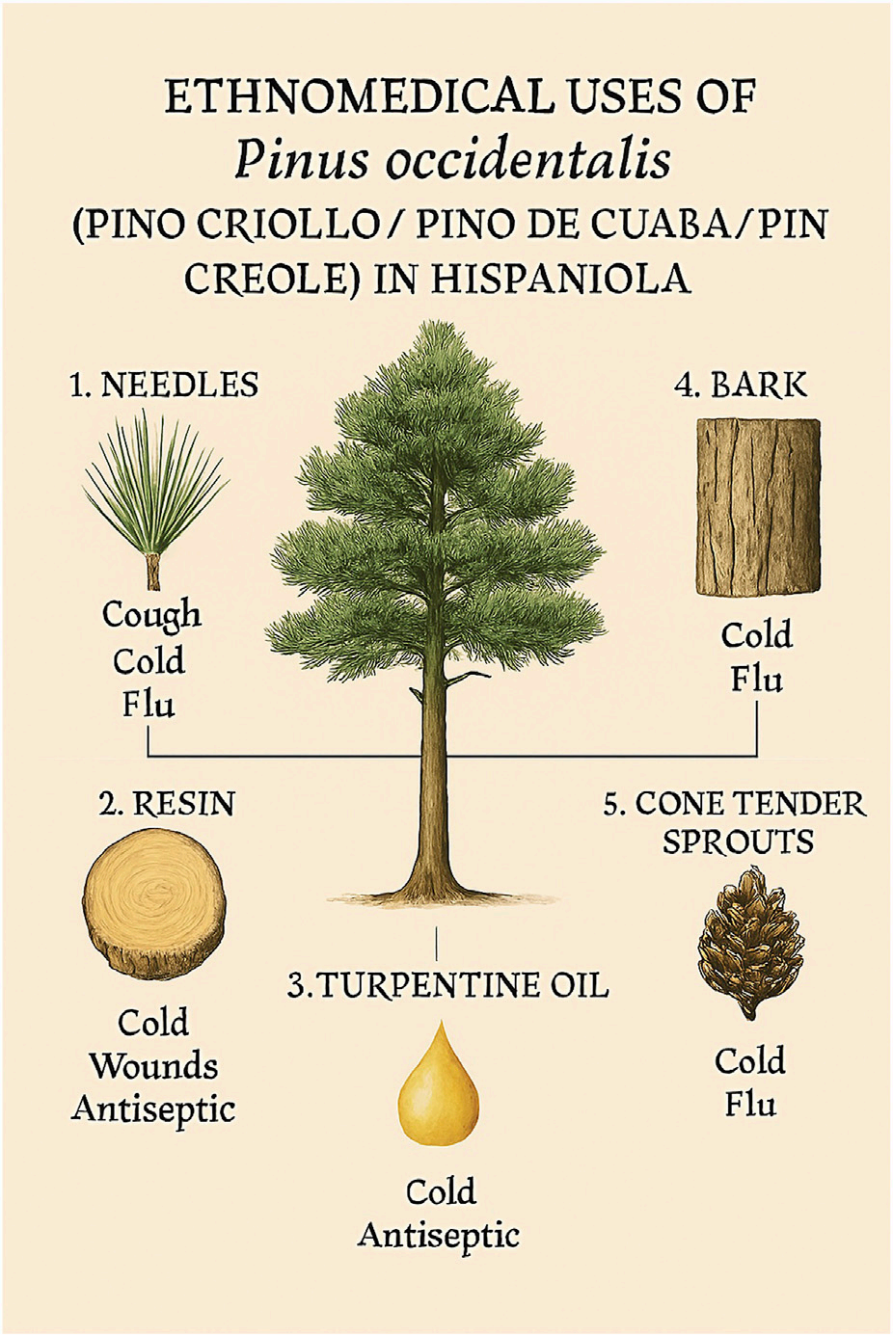
Ethnomedical healing map of *Pinus occidentalis* Swartz in Hispaniola.

Schulz Calvo (2014) reported that the “cuaba” soap, which is made with turpentine from the oily resin of *P. occidentalis*, has been used empirically as a skin antiseptic. The resin can be extracted and used as a raw material for the manufacture of turpentine, soap, and other purposes. Farmers use the tank or resinous wood for lighting and warmth. It is used in tea, mixed with other leaves, against the flu and colds. In Haiti, the resin and the essence of turpentine have been used against a series of ailments, both internal and external. Turpentine is a rubefacient; it is used in liniments and other pharmaceutical uses (Rouzier, 2014; Monografias, 2025; Thesnor et al., 2024). Still, there are only a few records on *P. occidentalis* medicinal uses, as compared to other *Pinus* spp., which demonstrates the need to carry out these studies (see Figure 3).

## 5 Ethnomedicine and chemical composition of *Pinus* spp.

### 5.1 Pine needles essential oils

EOs from pine needles are a complex mixture of more than 50 volatile compounds, and their reported medicinal effects depend on the *Pinus* sp., the location where they are grown, and the season of needle collection (Ioannou et al., 2014; Rabko et al., 2021). In folk medicine, EOs have been used for inhalations or aromatherapy for respiratory disorders (Rhind, 2018), and for topical skin applications for wounds and infections (Laub, 2018).

The major metabolites in pine EOs are β-pinene, camphene, α-pinene, sabinene, Δ^3^-carene, myrcene, α-terpineol, terpinolene, limonene, bornyl acetate, caryophyllene, terpinene-4-ol, γ-muurolene, phellandrene, α-terpinene, thujene, γ-terpinene, p-cymene, and germacrene D (Silori et al., 2019; Bhagat et al., 2018; Sharma et al., 2020). Most of the ethnobotanical and pharmacological effects of pine EOs are related to their antimicrobial activity (Ayaria and Romdhanea, 2020). It has been assumed that this antibacterial activity is related to specific monoterpene hydrocarbons, specifically to the content of α-pinene (Mirković et al., 2024). A higher antibacterial activity is related to the increased content of oxygenated terpenes, like α-terpineol and terpinene-4-ol, as in the case of *P. syvestris.* (Mitić et al., 2018). Pine needles’ EOs have been mainly used for treating infections and have demonstrated antibactericidal and antifungal effects. Antibacterial activities against *E. coli* and *S. aureus* have been reported for the EOs of three pine species (*P. thunbergii* Parl., *P. massoniana* Lamb., and *P. koraiensis* Siebold and Zucc. and these activities were correlated to the concentrations of *α*-pinene, *β*-pinene, and germacrene D in the oil (Liang et al., 2025). However, *P. massoniana* EO was the most active against *E. coli*, whereas *P. thunbergii* EO was active against *S. aureus*. These authors claimed that the antibacterial mechanism relies more on the synergic actions of EO metabolites than on a single component. *P. thunbergii* and *P. densiflora* Siebold and Zucc. EOs have also shown antibacterial activity against *K. pneumoniae, S. flexneri*, and *P. vulgaris* (Park and Lee, 2011). The needles’ EO from *P. peuce* Griseb. has shown antibacterial activity against *Strep. pneumonia*, *S. aureus, S. epidermidis, Strept. agalactiae, Acinetobacter* spp., and *Strept. pyogenes* (Karapandzova et al., 2011). Antioxidant and anti-inflammatory effects of pine needles’ EOs are the second most studied pharmacological effects. Koutsaviti et al. (2021) studied the chemical composition and the antioxidant activity of the needles’ EO obtained by hydrodistillation of 46 pine species. The highest antioxidant activity, measured by a chemiluminescence technique, was found in subsection *Australes* (the same subsection of *P. occidentalis*) for *P. attenuata* Lemmon (IC_50_ = 1.30 μg/mL) and *P. muricata* D. Don (IC_50_ = 1.60 μg/mL). Also, anti-inflammatory and analgesic effects of several pine needles’ EOs have been studied (Basholli-Salihu et al., 2017; Yang et al., 2021; Hajhashemi et al., 2021). These antioxidant, analgesic, and anti-inflammatory effects have been related to the needles’ polyphenol content. Among the identified polyphenols are catechin, quercetin, trifolin, taxifolin, and rhamnetin in *P. elliotii* (Zhang et al., 2024); flavonol glycosides, ferulic and p-coumaric acids, and catechin-derivated procyanidins, both dimers and trimers, in *P. nigra, P. sylvestris, P. mugo, and P. peuce* (Karapandzova et al., 2011); and catechin, epicatechin, five acids (gallic, vanillic, o- and p-coumaric, and ferulic), and dimers of catechin-epicatechin procyanidins in *P. eldarica* Silba (Sadeghi et al., 2014). According to this last report, polyphenol content in pine needles was 1.5 times less than in the pine bark extract.

One report about the ethnomedical use and the chemical composition of *P. occidentalis* needles EO was published in the 1990s (Zanoni et al., 1990). The authors claimed that the EO, alone or combined with *Pimenta racemosa* var. *ozua* (Urb. and Ekm.) (Landrum), is used as a cleaning agent, but this reference has not been documented. Needle samples were collected at Sierra de Bahoruco (see Figure 2), and the EO was obtained by hydrodistillation. GC/MS analysis identified twenty terpenoid metabolites, with four major ones: β-pinene (45%), germacrene D (22%), α-pinene (15%), and myrcene (9%). The high proportion of the sesquiterpene germacrene D was unusual compared to other pine EOs. In terms of a future research roadmap, ethnomedicinal uses of *P. occidentalis* needles have been identified as one of the gaps, along a more systematic study in regions of different altitudes, like Cordillera Central and Valle de Constanza in the Dominican Republic (see Figure 2). Further, experimental pharmacological and toxicological research, according to its ethnomedical uses, should be designed to complement a full assessment of *P. occidentalis* EO.

### 5.2 Pine bark resin and turpentine oil

The exuded pine bark resin is a mixture of terpenoids as the main metabolites (Neis et al., 2019). This resin is formed by a turpentine oil (a liquid volatile fraction, formed by mono- and sesquiterpenes), and rosin or gum (a solid non-volatile fraction, formed by diterpenes) (Zulak and Bohlmann, 2010). This gum has been extensively utilized due to its wide range of bioactivities and applications, and consists primarily of abietic- and pimaric-type resin acids (Yadav et al., 2016). Ethnomedical uses of *P. roxburghii* resin have included boils, heel cracks, and on either side just above the eye to remove swelling in the Indian Himalayas, and the *P. wallichiana* has been applied on heel cracks and other skin afflictions in the region of Uttar Pradesh (India) (Singh et al., 1990). The turpentine or wood oil from *P. roxburghii* resin is used in the Punjab region (Pakistan) as a nerve tonic and expectorant, as well as a remedy for treating burns and scalds, boils, cough, and gastric troubles (carminative) (Hussain et al., 2010). A review (Mercier et al., 2009) has summarized several ethnomedicinal uses of turpentine oil from *P. pinaster* in France, including antiparasitic, analgesic, revulsive, disinfectant (external use); balsamic, active on bronchial secretion and pulmonary and genito-urinary tract infections, hemostatic, dissolving gallstones, diuretic, antispasmodic, antirheumatic, and deworming activities. *P. nigra, P. brutia,* and *P. sylvestris* resins have been used in some regions of Turkey mainly for treating respiratory (cough, cold, flu, pneumonia, bronchitis, and asthma), and gastrointestinal (dyspepsia, ulcers, stomach spasm, and carminative) disorders by applying the resin externally in the chest or abdomen or by oral hot decoctions (Çakılcıoğlu et al., 2011; Çakır, 2017).

The turpentine oil of *P. occidentalis* has a significantly high amount of *α*-pinene (63.8%) (Mirov et al., 1962) as compared to other turpentines (Zavarin et al., 1968; Chalier et al., 2024). Other significant components were *β*-pinene (22.2%), and Δ^3^-carene (7.7%). Only one ethnomedical report was found regarding the use of *P. occidentalis* resin in hot decoctions, mixed with other medicinal herbs, for the treatment of colds and flu in the Dominican Republic, and the use of turpentine oil in Haiti as a rubefacient (Schulz Calvo, 2014). The main use of its resinous wood (“cuaba”) is for lighting. More studies regarding the ethnomedical uses of *P. occidentalis* gum and turpentine oils are needed, including the determination of their chemical composition, across different regions.

### 5.3 Cones and seeds

Pine cones have not been used extensively in traditional medicinal uses as compared to EOs and resin or turpentine oils (Mason, 2013). Two types of cones (female and male) can be found in pine trees, which produce seeds and pollen, respectively (Bramlett et al., 1977). The chemical composition of pine cone EOs has been published to some extent (Xu et al., 2012; Semerci et al., 2020; Latos-Brozio et al., 2021; Chammam et al., 2024). Cellulose, hemicellulose, and polysaccharides are the main components (85%–90%), with around 10%–15% of extractives including terpenoids and polyphenols as the main metabolites (Kumar et al., 2023). Cone essential oils obtained by hydrodistillation from *P. sylvestris, P. nigra, P. halepensis*, *P. pinea,* and *P. brutia* were characterized in Turkey (Tumen et al., 2010). α-pinene content varied from 15% (*P. sylvestris*) to 47% *(P. halepensis*), while a remarkably high content of limonene+β-phellandrene (70%) was found in the cone EO from *P. pinea,* and also of β-pinene (40%) in *P. brutia*. Cone EO from *P. armandii* Franch. (Southwest China) has α-pinene (21%) and limonene (16%) as the main metabolites, with a significant 33% of oxygenated monoterpenes (Yang et al., 2010). Unripe cones of *P. halepensis* have (+) catechin and procyanidins B3 and B6 as the main metabolites of the cone extract (Sakar et al., 1991).

One of the ethnomedical reports of *P. occidentalis* in the Dominican Republic is the use of tender sprouts of pine cones to treat cold and flu (Peguero, 2002). Major components identified in pine sprout tea from Korea include α-pinene, myrcene, β-thujene, terpinene-4-ol, and δ-cadinene (Kim and Chung, 2000), but similar studies on *P. occidentalis* have not been conducted.

### 5.4 Bark

The bark of *Pinus* spp. is a multilayered matrix whose complexity underlies a broad spectrum of biological and industrial applications (Szmechtyk and Małecka, 2024), and it accounts for around 15% of the tree’s weight (Kofujita et al., 1999; Murphy and Cown, 2015). The pine bark is removed before the chipping process and is used as boiler fuel or discarded as a waste residue (Neiva et al., 2020). Pine bark has been traditionally utilized in various cultures due to its rich polyphenolic composition and potential pharmacological benefits (Sharma et al., 2018; Singh et al., 2019; Sinha, 2019; Chupin et al., 2013; Gascon et al., 2018). Chemically, it can be divided into three main fractions: 1. structural polysaccharides, 2. lignin and polyphenolic extractives (phenolic acids, flavonoids, condensed tannins), and 3. terpenoid constituents (mono-, sesqui, and di-terpenes), most of them volatiles. Each fraction is characterized by key molecular building blocks whose structures and functional groups confer specific properties—from mechanical reinforcement to free-radical scavenging and several pharmacological effects.

#### 5.4.1 Structural polysaccharides

Cellulose and hemicelluloses account for around 50% of dry pine bark mass, depending on pine spp., soil, and climatic area (Feng et al., 2013; Ferreira-Santos et al., 2020; Fedorov and Ryazanova, 2021). Cellulose is a linear homopolysaccharide of β-D-glucose units linked by β-1,4-glycosidic bonds (repeating unit C_6_H_10_O_5_). In *P. pinaster* bark, holocellulose (cellulose + hemicelluloses) reaches 46.1% of dry weight, with α-cellulose ∼25.5% (Barros et al., 2023). Cellulose microfibrils provide tensile strength and serve as a scaffold for other polymers. Hemicelluloses are heteropolymers—principally xylan [→4)–β-D-Xylp–(1→]_n_ (C_5_H_8_O_4_)_n_ and glucomannan [→4)–β-D-Glcp–(1→ and →4)–β-D-Manp–(1→]_n_ (C_6_H_10_O_5_)_n_ (Srndovic, 2011). In *P. pinea* bark, monosaccharide profiling shows glucose 44.6%, mannose 18.2%, xylose 20.7%, galactose 7.6%, and arabinose 8.9% (Nunes et al., 1999). Hemicelluloses fill the space between cellulose fibrils, imparting flexibility. This group of chemical compounds has gained attention from the scientific community due to their immunomodulatory, antioxidant, and anti-inflammatory properties, which have been reviewed recently (Liu et al., 2025).

#### 5.4.2 Polyphenols

##### 5.4.2.1 Lignin

Lignin is an amorphous phenylpropanoid copolymer derived from three monolignols—p-coumaryl alcohol (C_9_H_10_O_2_), coniferyl alcohol (C_10_H_12_O_3_), and sinapyl alcohol (C_11_H_14_O_4_)—cross-linked via C–O–C (ether) and C–C bonds (Katahira et al., 2018). In *P. pinaster* bark, lignin (including non-hydrolyzable phenolic acids) comprises between 41% and 52% of dry weight (Nunes et al., 1996; Ferreira-Santos et al., 2020; Barros et al., 2023), and in *P. pinea,* ∼37.5% (Nunes et al., 1999). The abundance of guaiacyl units from coniferyl alcohol renders pine bark lignin particularly rich in methoxy functionalities, affecting its recalcitrance and antioxidant capacity (Neiva et al., 2020).

##### 5.4.2.2 Polyphenolic extractives

**FIGURE 4:**
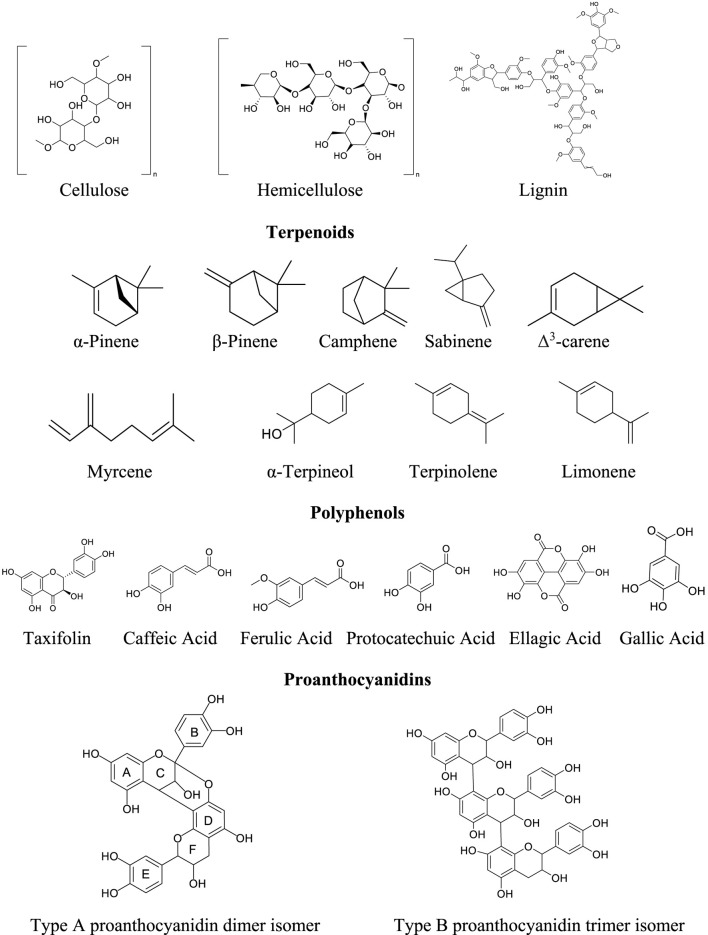
Chemical structures of bioactive components in pine tree parts (polysaccharides, lignans, terpenoids, polyphenols, and proanthocyanidins).

Accounting for 17%–19% of mass, polyphenolic extractives are responsible for antioxidant, anti-inflammatory, and vascular activities of pine bark extracts (Dridi and Bordenave, 2020). Four flavonoids (catechin, epicatechin, quercetin, and taxifolin) have been reported as the most abundant metabolites in pine bark extracts (Saleem et al., 2003; Yesil-Celiktas et al., 2009; Kim et al., 2018; Ramos et al., 2022; Szmechtyk and Małecka, 2024). These flavonoids possess a C_6_–C_3_–C_6_ skeleton with multiple hydroxyl groups, which grants a potent radical-scavenging activity. Their interactions with lignin-like phenolic structures may enhance cellular protection through synergistic antioxidant mechanisms (Mercado-Mercado et al., 2020). Polyphenolic acids include caffeic, ferulic, and protocatechuic acids as the most significant metabolites of polyphenolic extractives (Kim et al., 2025). Total phenolic acids in *Pinus* spp. bark extracts frequently exceed 50 mg.g^−1^ (GAE), as demonstrated in *P. patula* Schiede and *P. pinaster* bark, reflecting their richness in hydroxybenzoic and hydroxycinnamic acids (Cádiz-Gurrea et al., 2014; Sarria-Villa et al., 2017), and *P. radiata* for catechin and taxifolin (Bocalandro et al., 2012). Their ortho-dihydroxy (catechol) and methoxy phenolic structures facilitate hydrogen-donating antioxidant mechanisms and metal chelation. Chemical structures of these bark metabolites are shown in Figure 4.

Proanthocyanidins (PAs) are oligomeric chains of flavan-3-ol units (catechin/epicatechin). In *Pinus* spp. bark extracts, condensed tannins represent around 10% of anhydrous weight and more than 90% of total phenolics (Jerez et al., 2007). Structurally, B-type linkages (C4→C8 or C4→C6) between flavanol monomers yield dimers (procyanidin B1–B4) and higher oligomers (Yu et al., 2020). Their high molecular weight and multiple phenolic moieties enable protein precipitation (astringency) and robust antimicrobial action (Li et al., 2023). PAs are formed in the plant through a complex non-enzymatic process, in which conjugates of flavan-3-ols with glucose and cysteine may represent intermediates of PA biosynthesis (Dixon and Sarnala, 2020). Extension units attack the nucleophilic C8 (or less frequently C6) of a “starter” flavanol, yielding B-type linkages; A-type linkages arise when an interflavan carbocation also reacts with a phenolic oxygen at C7 (Qi et al., 2022). *P. pinaster* bark extract contains 65%–75% PAs, predominantly B-type dimers and trimers of epicatechin and catechin (Bayer and Högger, 2024). These PA fractions are credited with the extract’s vascular-protective and anti-inflammatory effects, through mechanisms such as endothelial nitric-oxide enhancement and NF-κB inhibition (Yang et al., 2018).

A summary of most of the reported metabolites of pine bark extracts (mono-, sesqui, di-and tri-terpenes, polyphenols, procyanidins, acids, long-chain alcohols, and sterols), including fifteen *Pinus* spp. (*P. pinaster* Aiton*, P. sylvestris* L.*, P. strobus* L.*, P. roxburghii* Sarg.*, P. nigra* J.F. Arnold*, P. eldarica* Silba*, P. pinea* L.*, P. densiflora* Siebold and Zucc.*, P. brutia* Ten.*, P. wallichiana* A.B. Jacks.*, P. gerardiana* Wall.*, P. halepensis* Mill.*, P. mugo* Turra*, P. radiata* D. Don, and *P. occidentalis* Swartz), are shown in a Supplementary Material. These data support the need to investigate the chemical composition of *P. occidentalis* tree parts in attempts to correlate observed medicinal effects with detected metabolites in the extracts. Figure 4 shows some of the chemical structures of the metabolites found in extracts of pine species (needles, cones, resin, and bark).

## 6 Conclusion

This manuscript represents the first comprehensive synthesis of the ethnomedical applications of *P. occidentalis* Swartz, systematically mapping its cultural and therapeutic significance across Hispaniola. The principal novelty of this work lies in its identification of a previously underappreciated pattern: while the resin’s and needles’ essential oil uses for respiratory ailments are documented, this analysis highlights consistent and diverse applications for treating dermatological, respiratory, and gastrointestinal conditions, areas that are less explored in the scientific literature. By consolidating disparate sources, this review establishes *P. occidentalis* not just as a cultural keystone species but as a significant reservoir of potential novel therapeutic metabolites, moving beyond its well-known resin to encompass the whole plant.

Despite these contributions, our findings must be interpreted in light of several limitations. The primary constraint is the inherent heterogeneity and qualitative nature of the available ethnobotanical sources, which often lack standardized data on preparation methods, dosages, and specific ailments. Our findings highlight a significant gap between traditional usage and scientific validation. For many documented applications, there is a lack of robust pharmacological and clinical studies, making it challenging to evaluate their efficacy, safety, and mechanisms of action.

## 7 Future perspectives

**FIGURE 5:**
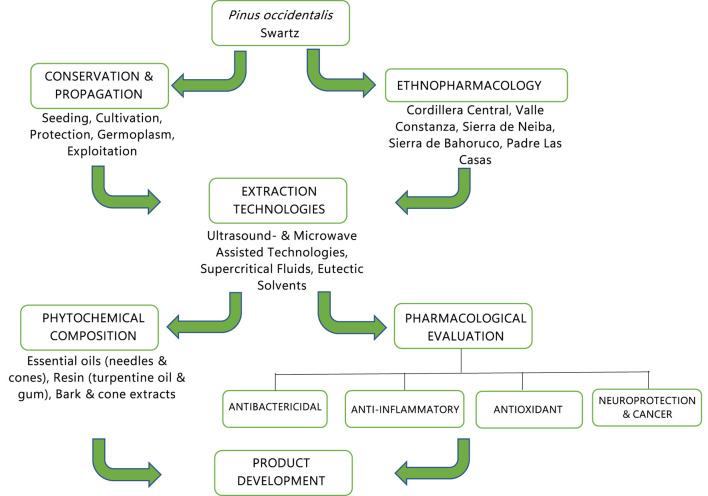
Proposal of a research roadmap flowchart for *P. occidentalis* Swartz.

More ethnopharmacological research is needed in Hispaniola about the uses of *P. occidentalis* parts (needles, resin, turpentine oil, bark, and cones) by native communities in both countries, Haiti and the Dominican Republic. From the fourteen *P. occidentalis* forests identified on the island (Figure 2), only a few have been studied from an ethnopharmacological point of view. This would be the first step for the research roadmap in the attempt to understand the cultural context of its medicinal use. Phytochemical characterization of *P. occidentalis* extracts needs intensive research since only two reports have characterized the needle’s essential oil and the turpentine oil in the past century. Research on *P. occidentalis* needles, bark, and cone extracts should prioritize the systematic elucidation of structure–activity relationships across the full spectrum of condensed tannin oligomers, terpenoid profiles, and lignin-derived phenolics to pinpoint the precise molecular features driving the pharmacological effects that must be studied to evidence the ethnomedical uses in Hispaniola. Parallel efforts must develop green-chemistry extraction and fractionation protocols—such as supercritical CO_2_ and deep-eutectic solvents—to maximize the yield of high-value proanthocyanidin dimers and trimers without degrading labile flavonols and resin acids. These results will expand the chemical library available for drug discovery, while biotechnological approaches, including plant cell cultures and microbial biotransformation, offer routes to sustainable, high-purity production of signature metabolites in *P. occidentalis.* The ultimate goal is to address the existing knowledge gaps of *P. occidentalis* and explore its potential applications in multi-modal therapeutic strategies—incorporating probiotics, low-dose pharmaceuticals, or other botanical extracts. By integrating advanced omics, green-chemistry protocols, and biotechnological production with large-scale human trials and strategic combination therapies, *P. occidentalis*, as endemic in Hispaniola, may be a source of novel metabolites for integrated therapies. Figure 5 shows the proposed research roadmap for *P. occidentalis* considering these results.
