# Supplementary material for: Ethnopharmacology of Pinus species with focus on the Hispaniola pine (Pinus occidentalis Swartz): evidence, gaps, and research roadmap
Source: Front Pharmacol. 2025 Nov 13;16:1680390. doi: 10.3389/fphar.2025.1680390 (PMC12657381; doi:10.3389/fphar.2025.1680390)
Supplement: Supplementary file 1 [file Table1.docx]

**Supplementary File.** Chemical composition of pine bark extracts determined by GC/MS, HPLC-UV, HPLC-DAD, and HPLC/MS. Fifteen *Pinus* spp. were included: *P. pinaster, P. sylvestris, P. strobus, P. roxburghii, P. nigra, P. eldarica, P. pinea, P. densiflora, P. brutia, P. wallichiana, P. gerardiana, P. halepensis, P. mugo, P. radiata,* and *P. occidentalis*

| **Compound** | **Pine sp** | **Extraction** | **Detected by** | | | | | **Content** | **References** |
| --- | --- | --- | --- | --- | --- | --- | --- | --- | --- |
|  |  |  | **GC/FID** | **GC/MS** | **HPLC/UV** | **HPLC/DAD** | **HPLC/MS** |  |  |
| **Volatiles** | | | | | | | | | |
| α-Pinene | *sylvestris* | UAE/MAE^a^ | X |  |  |  |  | 885-1826 μg/g bark | Nisca et al., 2021 |
|  |  | Solvent batch |  | X |  |  |  | 41.4 %^d^ | Hamad et al, 2019 |
|  |  | Steam dist. |  | X |  |  |  | 39.82% EO needles | Mirković et al., 2024 |
|  |  | Steam dist. |  | X |  |  |  | 37.86% EO cones | Mirković et al., 2024 |
|  | *nigra* | UAE/MAE^a^ | X |  |  |  |  | 2153-2412 μg/g bark | Nisca et al., 2021 |
|  |  | Steam dist. |  | X |  |  |  | 54.42% EO needles | Mirković et al., 2024 |
|  |  | Steam dist. |  | X |  |  |  | 40.0% EO cones | Mirković et al., 2024 |
|  | *eldarica* | Solvent batch |  | X |  |  |  | 24.6 % bark oil | Iravani & Zolfaghari, 2014 |
|  | *occidentalis* | Vacuum dist. | X^h^ |  |  |  |  | 63.8% turpentine oil | Mirov et al., 1962 |
|  |  | Steam dist. |  | X |  |  |  | 15.7% EO needles | Zanoni et al., 1990 |
|  | *halepensis* | Steam dist. |  | X |  |  |  | 17.02% EO needles | Mirković et al., 2024 |
|  |  | Steam dist. |  | X |  |  |  | 47.47% EO cones | Mirković et al., 2024 |
|  | *mugo* | Steam dist. |  | X |  |  |  | 1.89% EO needles | Mirković et al., 2024 |
|  |  | Steam dist. |  | X |  |  |  | 3.93% EO cones | Mirković et al., 2024 |
|  | *roxburghii* | Steam dist. |  | X |  |  |  | 15.4% EO needles | Baghat et al., 2018 |
|  |  | Steam dist. |  | X |  |  |  | 12.7% EO needles | Sharma et al., 2020 |
|  |  | Steam dist. |  | X |  |  |  | 3.4% bark oil | Sharma et al., 2020 |
|  | *wallichiana* | Steam dist. |  | X |  |  |  | 36.0% EO needles | Sharma et al., 2020 |
|  |  | Steam dist. |  | X |  |  |  | 30.2% bark oil | Sharma et al., 2020 |
|  | *gerardiana* | Steam dist. |  | X |  |  |  | 46.8% EO needles | Sharma et al., 2020 |
|  |  | Steam dist. |  | X |  |  |  | 16.3% EO bark oil | Sharma et al., 2020 |
| β-Pinene | *sylvestris* | UAE/MAE^a^ | X |  |  |  |  | 3422-3518 μg/g bark | Nisca et al., 2021 |
|  |  | Solvent batch |  | X |  |  |  | 6.9%^d^ | Hamad et al, 2019 |
|  |  | Steam dist. |  | X |  |  |  | 6.02% EO needles | Mirković et al., 2024 |
|  |  | Steam dist. |  | X |  |  |  | 6.78% EO cones | Mirković et al., 2024 |

Table (continued)

| **Compound** | | **Pine sp** | **Extraction** | **Detected by** | | | | | **Content** | **References** |
| --- | --- | --- | --- | --- | --- | --- | --- | --- | --- | --- |
|  |  |  |  | **GC/FID** | **GC/MS** | **HPLC/UV** | **HPLC/DAD** | **HPLC/MS** |  |  |
| β-Pinene | *nigra* | | UAE/MAE^a^ | X |  |  |  |  | 3239-3794 μg/g bark | Nisca et al., 2021 |
|  |  |  | Steam dist. |  | X |  |  |  | 3.59% EO needles | Mirković et al., 2024 |
|  |  |  | Steam dist. |  | X |  |  |  | 2.49% EO cones | Mirković et al., 2024 |
|  | *eldarica* | | Solvent batch |  | X |  |  |  | 0.7 % bark oil | Iravani & Zolfaghari, 2014 |
|  | *roxburghii* | | Steam dist. |  | X |  |  |  | 2.46% EO needles | Baghat et al., 2018 |
|  | *occidentalis* | | Vacuum dist. | X^h^ |  |  |  |  | 22.2% turpentine oil | Mirov et al., 1962 |
|  |  |  | Steam dist. |  | X |  |  |  | 45.4% EO needles | Zanoni et al., 1990 |
|  | *halepensis* | | Steam dist. |  | X |  |  |  | 3.44% EO needles | Mirković et al., 2024 |
|  |  |  | Steam dist. |  | X |  |  |  | 3.85% EO cones | Mirković et al., 2024 |
|  | *mugo* | | Steam dist. |  | X |  |  |  | 6.56% EO needles | Mirković et al., 2024 |
|  |  |  | Steam dist. |  | X |  |  |  | 2.28% EO cones | Mirković et al., 2024 |
| Δ^3^-Carene | *sylvestris* | | UAE/MAE^a^ | X |  |  |  |  | 68.8 – 91.0 μg/g bark | Nisca et al., 2021 |
|  |  |  | Steam dist. |  | X |  |  |  | 0.07% EO cones | Mirković et al., 2024 |
|  | *nigra* | | UAE/MAE^a^ | X |  |  |  |  | 80.9 – 84.2 μg/g bark | Nisca et al., 2021 |
|  |  |  | Steam dist. |  | X |  |  |  | 0.02% EO needles | Mirković et al., 2024 |
|  | *eldarica* | | Solvent batch |  | X |  |  |  | 10.7 % bark oil | Iravani & Zolfaghari, 2014 |
|  | *occidentalis* | | Vacuum dist. | X^h^ |  |  |  |  | 7.7% turpentine oil | Mirov et al., 1962 |
|  | *halepensis* | | Steam dist. |  | X |  |  |  | 5.10% EO cones | Mirković et al., 2024 |
|  | *mugo* | | Steam dist. |  | X |  |  |  | 23.36% EO needles | Mirković et al., 2024 |
|  |  |  | Steam dist. |  | X |  |  |  | 19.95% EO cones | Mirković et al., 2024 |
|  | *roxburghii* | | Steam dist. |  | X |  |  |  | 17.8% EO needles | Sharma et al., 2020 |
|  |  |  | Steam dist. |  | X |  |  |  | 22.5% bark oil | Sharma et al., 2020 |
|  | *gerardiana* | | Steam dist. |  | X |  |  |  | 24.0% EO needles | Sharma et al., 2020 |
|  |  |  | Steam dist. |  | X |  |  |  | 31.1% EO bark oil | Sharma et al., 2020 |
| α-Phellandrene | *sylvestris* | | UAE/MAE^a^ | X |  |  |  |  | 7.1 – 11.1 μg/g bark | Nisca et al., 2021 |
|  |  |  | Steam dist. |  | X |  |  |  | 0.04% EO needles | Mirković et al., 2024 |
|  | *nigra* | | UAE/MAE^a^ | X |  |  |  |  | 9.8 – 10.2 μg/g bark | Nisca et al., 2021 |
|  |  |  | Steam dist. |  | X |  |  |  | 0.03% EO needles | Mirković et al., 2024 |

Table (continued)

| **Compound** | | **Pine sp** | **Extraction** | **Detected by** | | | | | **Content** | **References** |
| --- | --- | --- | --- | --- | --- | --- | --- | --- | --- | --- |
|  |  |  |  | **GC/FID** | **GC/MS** | **HPLC/UV** | **HPLC/DAD** | **HPLC/MS** |  |  |
| α-Phellandrene | *mugo* | | Steam dist. |  | X |  |  |  | 0.19% EO needles | Mirković et al., 2024 |
|  |  |  | Steam dist. |  | X |  |  |  | 0.12% EO cones | Mirković et al., 2024 |
|  | *gerardiana* | | Steam dist. |  | X |  |  |  | 3.9% EO needles | Sharma et al., 2020 |
|  |  |  | Steam dist. |  | X |  |  |  | 5.9% EO bark oil | Sharma et al., 2020 |
| β-Phellandrene | *sylvestris* | | Steam dist. |  | X |  |  |  | 2.23% EO needles | Mirković et al., 2024 |
|  | *halepensis* | | Steam dist. |  | X |  |  |  | 2.81% EO needles | Mirković et al., 2024 |
|  | *mugo* | | Steam dist. |  | X |  |  |  | 5.75% EO needles | Mirković et al., 2024 |
|  |  |  | Steam dist. |  | X |  |  |  | 7.33% EO cones | Mirković et al., 2024 |
|  | *roxburghii* | | Steam dist. |  | X |  |  |  | 39.63% EO needles | Baghat et al., 2018 |
|  | *occidentalis* | | Steam dist. |  | X |  |  |  | 0.81% EO needles | Zanoni et al., 1990 |
| α-Thujene | *sylvestris* | | Steam dist. |  | X |  |  |  | 0.35% EO needles | Mirković et al., 2024 |
|  | *mugo* | | Steam dist. |  | X |  |  |  | 1.62% EO needles | Mirković et al., 2024 |
|  |  |  | Steam dist. |  | X |  |  |  | 0.09% EO cones | Mirković et al., 2024 |
|  | *halepensis* | | Steam dist. |  | X |  |  |  | 0.30% EO needles | Mirković et al., 2024 |
|  | *roxburghii* | | Steam dist. |  | X |  |  |  | 1.67% EO needles | Baghat et al., 2018 |
| Limonene | *sylvestris* | | UAE/MAE^a^ | X |  |  |  |  | 74.2 – 94.3 μg/g bark | Nisca et al., 2021 |
|  |  |  | Solvent batch |  | X |  |  |  | 4.5 %^d^ | Hamad et al, 2019 |
|  |  |  | Steam dist. |  | X |  |  |  | 6.92% EO cones | Mirković et al., 2024 |
|  | *nigra* | | UAE/MAE^a^ | X |  |  |  |  | 97.2 – 109.9 μg/g | Nisca et al., 2021 |
|  |  |  | Steam dist. |  | X |  |  |  | 2.57% EO needles | Mirković et al., 2024 |
|  |  |  | Steam dist. |  | X |  |  |  | 0.96% EO cones | Mirković et al., 2024 |
|  | *eldarica* | | Solvent batch |  | X |  |  |  | 1.5 % bark oil | Iravani & Zolfaghari, 2014 |
|  | *halepensis* | | Steam dist. |  | X |  |  |  | 5.98% EO cones | Mirković et al., 2024 |
|  | *roxburghii* | | Steam dist. |  | X |  |  |  | 1.19% EO needles | Baghat et al., 2018 |
|  |  |  | Steam dist. |  | X |  |  |  | 2.5% EO needles | Sharma et al., 2020 |
|  |  |  | Steam dist. |  | X |  |  |  | 4.9% bark oil | Sharma et al., 2020 |
|  | *wallichiana* | | Steam dist. |  | X |  |  |  | 2.1% EO needles | Sharma et al., 2020 |
|  |  |  | Steam dist. |  | X |  |  |  | 3.2% bark oil | Sharma et al., 2020 |
|  | *occidentalis* | | Steam dist. |  | X |  |  |  | 0.80% EO | Zanoni et al., 1990 |

Table (continued)

| **Compound** | | **Pine sp** | **Extraction** | **Detected by** | | | | | **Content** | **References** |
| --- | --- | --- | --- | --- | --- | --- | --- | --- | --- | --- |
|  |  |  |  | **GC/FID** | **GC/MS** | **HPLC/UV** | **HPLC/DAD** | **HPLC/MS** |  |  |
| Sabinene | *sylvestris* | | UAE/MAE^a^ | X |  |  |  |  | 31.3 -37.3 μg/g bark | Nisca et al., 2021 |
|  | *nigra* | | UAE/MAE^a^ | X |  |  |  |  | 49.1 – 59.9 μg/g bark | Nisca et al., 2021 |
|  |  |  | Steam dist. |  | X |  |  |  | 1.69% EO needles | Mirković et al., 2024 |
|  |  |  | Steam dist. |  | X |  |  |  | 0.66% EO cones | Mirković et al., 2024 |
|  | *halepensis* | | Steam dist. |  | X |  |  |  | 2.49% EO needles | Mirković et al., 2024 |
|  | *occidentalis* | | Steam dist. |  | X |  |  |  | 0.08% EO | Zanoni et al., 1990 |
| β-Selinene | *sylvestris* | | Steam dist. |  | X |  |  |  | 0.56% EO needles | Mirković et al., 2024 |
|  | *eldarica* | | Solvent batch |  | X |  |  |  | 2.0 % bark oil | Iravani & Zolfaghari, 2014 |
| Myrcene | *sylvestris* | | UAE/MAE^a^ | X |  |  |  |  | 112.7 – 156.2 μg/g | Nisca et al., 2021 |
|  |  |  | Solvent batch |  | X |  |  |  | 3.2 %^d^ | Hamad et al, 2019 |
|  |  |  | Steam dist. |  | X |  |  |  | 1.69% EO needles | Mirković et al., 2024 |
|  |  |  | Steam dist. |  | X |  |  |  | 13.78% EO cones | Mirković et al., 2024 |
|  | *nigra* | | UAE/MAE^a^ | X |  |  |  |  | 136.3 – 139.9 μg/g | Nisca et al., 2021 |
|  |  |  | Steam dist. |  | X |  |  |  | 1.22% EO needles | Mirković et al., 2024 |
|  | *halepensis* | | Steam dist. |  | X |  |  |  | 24.65% EO needles | Mirković et al., 2024 |
|  |  |  | Steam dist. |  | X |  |  |  | 14.61% EO cones | Mirković et al., 2024 |
|  | *mugo* | | Steam dist. |  | X |  |  |  | 2.41% EO needles | Mirković et al., 2024 |
|  |  |  | Steam dist. |  | X |  |  |  | 1.38% EO cones | Mirković et al., 2024 |
|  | *roxburghii* | | Steam dist. |  | X |  |  |  | 2.1% EO needles | Sharma et al., 2020 |
|  |  |  | Steam dist. |  | X |  |  |  | 2.1% bark oil | Sharma et al., 2020 |
|  | *wallichiana* | | Steam dist. |  | X |  |  |  | 1.3% bark oil | Sharma et al., 2020 |
|  | *gerardiana* | | Steam dist. |  | X |  |  |  | 0.7% EO needles | Sharma et al., 2020 |
|  |  |  | Steam dist. |  | X |  |  |  | 1.2% bark oil | Sharma et al., 2020 |
|  | *occidentalis* | | Steam dist. |  | X |  |  |  | 9.41% EO | Zanoni et al., 1990 |
| γ-Terpinene | *sylvestris* | | Steam dist. |  | X |  |  |  | 0.05% EO needles | Mirković et al., 2024 |
|  | *eldarica* | | Solvent batch |  | X |  |  |  | 0.4 % bark oil | Iravani & Zolfaghari, 2014 |
|  | *halepensis* | | Steam dist. |  | X |  |  |  | 0.13% EO needles | Mirković et al., 2024 |
|  |  |  | Steam dist. |  | X |  |  |  | 0.04% EO cones | Mirković et al., 2024 |

Table (continued)

| **Compound** | | **Pine sp** | **Extraction** | **Detected by** | | | | | **Content** | **References** |
| --- | --- | --- | --- | --- | --- | --- | --- | --- | --- | --- |
|  |  |  |  | **GC/FID** | **GC/MS** | **HPLC/UV** | **HPLC/DAD** | **HPLC/MS** |  |  |
| γ-Terpinene | *mugo* | | Steam dist. |  | X |  |  |  | 0.25% EO needles | Mirković et al., 2024 |
|  |  |  | Steam dist. |  | X |  |  |  | 0.17% EO cones | Mirković et al., 2024 |
|  | *roxburghii* | | Steam dist. |  | X |  |  |  | 1.69% EO needles | Baghat et al., 2018 |
|  |  |  | Steam dist. |  | X |  |  |  | 50.9% EO needles | Sharma et al., 2020 |
| Tricyclene | | *sylvestris* | UAE/MAE^a^ | X |  |  |  |  | 7.9 – 8.2 μg/g bark | Nisca et al., 2021 |
|  |  |  | Steam dist. |  | X |  |  |  | 0.66% EO needles | Mirković et al., 2024 |
|  |  | *nigra* | UAE/MAE^a^ | X |  |  |  |  | 6.3 – 9. 3 μg/g bark | Nisca et al., 2021 |
|  |  |  | Steam dist. |  | X |  |  |  | 0.21% EO needles | Mirković et al., 2024 |
|  |  | *mugo* | Steam dist. |  | X |  |  |  | 0.48% EO needles | Mirković et al., 2024 |
|  |  | *halepensis* | Steam dist. |  | X |  |  |  | 0.08% EO needles | Mirković et al., 2024 |
|  |  |  | Steam dist. |  | X |  |  |  | 0.07% EO cones | Mirković et al., 2024 |
|  |  | *occidentalis* | Steam dist. |  | X |  |  |  | 0.06% EO | Zanoni et al., 1990 |
| Cembrene | | *sylvestris* | Solvent batch |  | X |  |  |  | 6.3 %^d^ | Hamad et al, 2019 |
|  |  | *nigra* | Steam dist. |  | X |  |  |  | 0.70% EO needles | Mirković et al., 2024 |
|  |  |  | Steam dist. |  | X |  |  |  | 0.44% EO cones | Mirković et al., 2024 |
|  |  | *mugo* | Steam dist. |  | X |  |  |  | 0.18% EO needles | Mirković et al., 2024 |
|  |  |  | Steam dist. |  | X |  |  |  | 0.11% EO cones | Mirković et al., 2024 |
|  |  | *halepensis* | Steam dist. |  | X |  |  |  | 1.05% EO needles | Mirković et al., 2024 |
| Camphene | | *sylvestris* | Steam dist. |  | X |  |  |  | 3.08% EO needles | Mirković et al., 2024 |
|  |  |  | Steam dist. |  | X |  |  |  | 0.74% EO cones | Mirković et al., 2024 |
|  |  | *nigra* | Steam dist. |  | X |  |  |  | 1.16% EO needles | Mirković et al., 2024 |
|  |  |  | Steam dist. |  | X |  |  |  | 0.69% EO cones | Mirković et al., 2024 |
|  |  | *mugo* | Steam dist. |  | X |  |  |  | 1.41% EO needles | Mirković et al., 2024 |
|  |  |  | Steam dist. |  | X |  |  |  | 1.21% EO cones | Mirković et al., 2024 |
|  |  | *halepensis* | Steam dist. |  | X |  |  |  | 0.18% EO needles | Mirković et al., 2024 |
|  |  |  | Steam dist. |  | X |  |  |  | 0.65% EO cones | Mirković et al., 2024 |
|  |  | *eldarica* | Solvent batch |  | X |  |  |  | 1.1 % bark oil | Iravani & Zolfaghari, 2014 |
|  |  | *roxburghii* | Steam dist. |  | X |  |  |  | 0.3% EO needles | Sharma et al., 2020 |
|  |  |  | Steam dist. |  | X |  |  |  | 0.6% bark oil | Sharma et al., 2020 |

Table (continued)

| **Compound** | **Pine sp** | **Extraction** | **Detected by** | | | | | **Content** | **References** |
| --- | --- | --- | --- | --- | --- | --- | --- | --- | --- |
|  |  |  | **GC/FID** | **GC/MS** | **HPLC/UV** | **HPLC/DAD** | **HPLC/MS** |  |  |
| Camphene | *wallichiana* | Steam dist. |  | X |  |  |  | 2.4% bark oil | Sharma et al., 2020 |
|  | *gerardiana* | Steam dist. |  | X |  |  |  | 0.5% EO needles | Sharma et al., 2020 |
|  |  | Steam dist. |  | X |  |  |  | 1.0% bark oil | Sharma et al., 2020 |
|  | *occidentalis* | Vacuum dist. | X^h^ |  |  |  |  | 0.2% turpentine oil | Mirov et al., 1962 |
|  |  | Steam dist. |  | X |  |  |  | 0.41% EO | Zanoni et al., 1990 |
| Verbenene | *nigra* | Steam dist. |  | X |  |  |  | 0.24% EO needles | Mirković et al., 2024 |
|  | *eldarica* | Solvent batch |  | X |  |  |  | 1.3 % bark oil | Iravani & Zolfaghari, 2014 |
| Mesytelene | *eldarica* | Solvent batch |  | X |  |  |  | 0.7 % bark oil | Iravani & Zolfaghari, 2014 |
| p-Cymene | *sylvestris* | Steam dist. |  | X |  |  |  | 0.22% EO needles | Mirković et al., 2024 |
|  | *nigra* | Steam dist. |  | X |  |  |  | 0.05% EO needles | Mirković et al., 2024 |
|  | *mugo* | Steam dist. |  | X |  |  |  | 0.19% EO needles | Mirković et al., 2024 |
|  |  | Steam dist. |  | X |  |  |  | 0.31% EO cones | Mirković et al., 2024 |
|  | *halepensis* | Steam dist. |  | X |  |  |  | 0.09% EO cones | Mirković et al., 2024 |
|  | *eldarica* | Solvent batch |  | X |  |  |  | 0.3 % bark oil | Iravani & Zolfaghari, 2014 |
|  | *occidentalis* | Steam dist. |  | X |  |  |  | 0.06% EO needless | Zanoni et al., 1990 |
| m-Cymene | *eldarica* | Solvent batch |  | X |  |  |  | 1.9 % bark oil | Iravani & Zolfaghari, 2014 |
| Terpinolene | *nigra* | Steam dist. |  | X |  |  |  | 0.34% EO needles | Mirković et al., 2024 |
|  | *mugo* | Steam dist. |  | X |  |  |  | 3.48% EO needles | Mirković et al., 2024 |
|  |  | Steam dist. |  | X |  |  |  | 1.57% EO cones | Mirković et al., 2024 |
|  | *halepensis* | Steam dist. |  | X |  |  |  | 2.06% EO needles | Mirković et al., 2024 |
|  |  | Steam dist. |  | X |  |  |  | 0.38% EO cones | Mirković et al., 2024 |
|  | *roxburghii* | Steam dist. |  | X |  |  |  | 11.78% EO needles | Baghat et al., 2018 |
|  | *occidentalis* | Steam dist. |  | X |  |  |  | 0.23% EO needles | Zanoni et al., 1990 |
| α-Terpineol | *sylvestris* | Solvent batch |  | X |  |  |  | 4.8 %^d^ | Hamad et al, 2019 |
|  |  | Steam dist. |  | X |  |  |  | 0.07% EO needles | Mirković et al., 2024 |
|  |  | Steam dist. |  | X |  |  |  | 0.13% EO cones | Mirković et al., 2024 |
|  | *pinaster* | Soxhlet |  | X |  |  |  | 13 μg/g bark^e^ | Sousa et al, 2018 |
|  | *eldarica* | Solvent batch |  | X |  |  |  | 1.9 % bark oil | Iravani & Zolfaghari, 2014 |
|  | *nigra* | Steam dist. |  | X |  |  |  | 0.06% EO needles | Mirković et al., 2024 |

Table (continued)

| **Compound** | **Pine sp** | **Extraction** | **Detected by** | | | | | **Content** | **References** |
| --- | --- | --- | --- | --- | --- | --- | --- | --- | --- |
|  |  |  | **GC/FID** | **GC/MS** | **HPLC/UV** | **HPLC/DAD** | **HPLC/MS** |  |  |
| α-Terpineol | *halepensis* | Steam dist. |  | X |  |  |  | 0.24% EO cones | Mirković et al., 2024 |
|  | *occidentalis* | Steam dist. |  | X |  |  |  | 0.69% EO needles | Zanoni et al., 1990 |
| Camphor | *sylvestris* | Steam dist. |  | X |  |  |  | 0.03% EO needles | Mirković et al., 2024 |
|  | *pinaster* | Soxhlet |  | X |  |  |  | 2 μg/g bark^e^ | Sousa et al, 2018 |
|  | *eldarica* | Solvent batch |  | X |  |  |  | 0.4 % bark oil | Iravani & Zolfaghari, 2014 |
| Pinocarveol | *sylvestris* | Steam dist. |  | X |  |  |  | 0.09% EO needles | Mirković et al., 2024 |
|  |  | Steam dist. |  | X |  |  |  | 0.21% EO cones | Mirković et al., 2024 |
|  | *nigra* | Steam dist. |  | X |  |  |  | 0.05% EO needles | Mirković et al., 2024 |
|  | *mugo* | Steam dist. |  | X |  |  |  | 0.06% EO cones | Mirković et al., 2024 |
|  | *halepensis* | Steam dist. |  | X |  |  |  | 0.06% EO cones | Mirković et al., 2024 |
|  | *eldarica* | Solvent batch |  | X |  |  |  | 2.4 % bark oil | Iravani & Zolfaghari, 2014 |
|  | *occidentalis* | Steam dist. |  | X |  |  |  | 0.16% EO needles | Zanoni et al., 1990 |
| Pinocarvone | *sylvestris* | Steam dist. |  | X |  |  |  | 0.09% EO needles | Mirković et al., 2024 |
|  | *pinaster* | Soxhlet |  | X |  |  |  | 6 μg/g bark^e^ | Sousa et al, 2018 |
|  | *eldarica* | Solvent batch |  | X |  |  |  | 0.9 % bark oil | Iravani & Zolfaghari, 2014 |
|  | *occidentalis* | Steam dist. |  | X |  |  |  | 0.08% EO needles | Zanoni et al., 1990 |
| Verbenone | *eldarica* | Solvent batch |  | X |  |  |  | 0.7 % bark oil | Iravani & Zolfaghari, 2014 |
| Geraniol | *pinaster* | Soxhlet |  | X |  |  |  | 4 μg/g bark^e^ | Sousa et al, 2018 |
| Linalol | *occidentalis* | Vacuum dist. | X^h^ |  |  |  |  | 7.7% turpentine oil | Mirov et al., 1962 |
| Fenchol | *roxburghii* | Steam dist. |  | X |  |  |  | 0.37% EO needles | Baghat et al., 2018 |
|  | *occidentalis* | Steam dist. |  | X |  |  |  | 0.06% EO needles | Zanoni et al., 1990 |
| Borneol | *sylvestris* | Steam dist. |  | X |  |  |  | 0.08% EO needles | Mirković et al., 2024 |
|  |  | Steam dist. |  | X |  |  |  | 0.04% EO cones | Mirković et al., 2024 |
|  | *pinaster* | Soxhlet |  | X |  |  |  | 10 μg/g bark^e^ | Sousa et al, 2018 |
|  | *halepensis* | Steam dist. |  | X |  |  |  | 0.04% EO cones | Mirković et al., 2024 |
|  | *eldarica* | Solvent batch |  | X |  |  |  | 1.6 % bark oil | Iravani & Zolfaghari, 2014 |
|  | *occidentalis* | Steam dist. |  | X |  |  |  | trace EO needles | Zanoni et al., 1990 |
| Myrtenol | *pinaster* | Soxhlet |  | X |  |  |  | 4 μg/g bark^e^ | Sousa et al, 2018 |
|  | *sylvestris* | Steam dist. |  | X |  |  |  | 0.17% EO cones | Mirković et al., 2024 |

Table (continued)

| **Compound** | **Pine sp** | **Extraction** | **Detected by** | | | | | **Content** | **References** |
| --- | --- | --- | --- | --- | --- | --- | --- | --- | --- |
|  |  |  | **GC/FID** | **GC/MS** | **HPLC/UV** | **HPLC/DAD** | **HPLC/MS** |  |  |
| Myrtenol | *nigra* | Steam dist. |  | X |  |  |  | 0.04% EO needles | Mirković et al., 2024 |
| cis-Myrtanol | *eldarica* | Solvent batch |  | X |  |  |  | 0.7 % bark oil | Iravani & Zolfaghari, 2014 |
| α-Phellandren-8-ol | *eldarica* | Solvent batch |  | X |  |  |  | 0.5 % bark oil | Iravani & Zolfaghari, 2014 |
| Terpinen-4-ol | *mugo* | Steam dist. |  | X |  |  |  | 0.56% EO needles | Mirković et al., 2024 |
|  | *roxburghii* | Steam dist. |  | X |  |  |  | 2.64% EO needles | Baghat et al., 2018 |
|  | *occidentalis* | Steam dist. |  | X |  |  |  | 0.09% EO needles | Zanoni et al., 1990 |
| Terpin | *pinaster* | Soxhlet |  | X |  |  |  | 16 μg/g bark^e^ | Sousa et al, 2018 |
| Myrtenal | *sylvestris* | Steam dist. |  | X |  |  |  | 0.18% EO cones | Mirković et al., 2024 |
|  | *eldarica* | Solvent batch |  | X |  |  |  | 3.1 % bark oil | Iravani & Zolfaghari, 2014 |
|  | *occidentalis* | Steam dist. |  | X |  |  |  | 0.19% EO needles | Zanoni et al., 1990 |
| **Sesquiterpenes** | | | | | | | | | |
| Longifolene | *sylvestris* | Steam dist. |  | X |  |  |  | 1.26% EO cones | Mirković et al., 2024 |
|  | *nigra* | Steam dist. |  | X |  |  |  | 0.05% EO cones | Mirković et al., 2024 |
|  | *mugo* | Steam dist. |  | X |  |  |  | 0.09% EO cones | Mirković et al., 2024 |
|  | *eldarica* | Solvent batch |  | X |  |  |  | 0.5 % bark oil | Iravani & Zolfaghari, 2014 |
|  | *roxburghii* | Steam dist. |  | X |  |  |  | 4.7% bark oil | Sharma et al., 2020 |
|  | *gerardiana* | Steam dist. |  | X |  |  |  | 1.0% bark oil | Sharma et al., 2020 |
|  | *occidentalis* | Vacuum dist. | X^h^ |  |  |  |  | 0.5% turpentine oil | Mirov et al., 1962 |
| Longifolone | *pinaster* | Soxhlet |  | X |  |  |  | 21 μg/g bark^e^ | Sousa et al, 2018 |
|  | *pinea* | Soxhlet |  | X |  |  |  | 2 μg/g bark^e^ | Sousa et al, 2018 |
| Isolongifol | *pinaster* | Soxhlet |  | X |  |  |  | 4 μg/g bark^e^ | Sousa et al, 2018 |
| β-Caryophyllene | *pinaster* | Soxhlet |  | X |  |  |  | 7 μg/g bark^e^ | Sousa et al, 2018 |
|  | *sylvestris* | Steam dist. |  | X |  |  |  | 4.81% EO needles | Mirković et al., 2024 |
|  |  | Steam dist. |  | X |  |  |  | 9.13% EO cones | Mirković et al., 2024 |
|  | *nigra* | Steam dist. |  | X |  |  |  | 8.50% EO needles | Mirković et al., 2024 |
|  |  | Steam dist. |  | X |  |  |  | 14.00% EO cones | Mirković et al., 2024 |
|  | *mugo* | Steam dist. |  | X |  |  |  | 5.94% EO needles | Mirković et al., 2024 |
|  |  | Steam dist. |  | X |  |  |  | 21.07% EO cones | Mirković et al., 2024 |

Table (continued)

| **Compound** | **Pine sp** | **Extraction** | **Detected by** | | | | | **Content** | **References** |
| --- | --- | --- | --- | --- | --- | --- | --- | --- | --- |
|  |  |  | **GC/FID** | **GC/MS** | **HPLC/UV** | **HPLC/DAD** | **HPLC/MS** |  |  |
| β-Caryophyllene | *halepensis* | Steam dist. |  | X |  |  |  | 24.69% EO needles | Mirković et al., 2024 |
|  |  | Steam dist. |  | X |  |  |  | 11.70% EO cones | Mirković et al., 2024 |
|  | *pinea* | Soxhlet |  | X |  |  |  | 3 μg/g bark^e^ | Sousa et al, 2018 |
|  | *eldarica* | Solvent batch |  | X |  |  |  | 7.9 % bark oil | Iravani & Zolfaghari, 2014 |
|  | *roxburghii* | Steam dist. |  | X |  |  |  | 4.33% EO needles | Baghat et al., 2018 |
|  |  | Steam dist. |  | X |  |  |  | 19.5% EO needles | Sharma et al., 2020 |
|  | *gerardiana* | Steam dist. |  | X |  |  |  | 9.1% EO needles | Sharma et al., 2020 |
|  |  | Steam dist. |  | X |  |  |  | 1.0% bark oil | Sharma et al., 2020 |
| Caryophyllene oxide | *sylvestris* | Steam dist. |  | X |  |  |  | 0.66% EO cones | Mirković et al., 2024 |
|  | *nigra* | Steam dist. |  | X |  |  |  | 0.42% EO needles | Mirković et al., 2024 |
|  |  | Steam dist. |  | X |  |  |  | 0.55% EO cones | Mirković et al., 2024 |
|  | *mugo* | Steam dist. |  | X |  |  |  | 0.67% EO needles | Mirković et al., 2024 |
|  |  | Steam dist. |  | X |  |  |  | 0.78% EO cones | Mirković et al., 2024 |
|  | *halepensis* | Steam dist. |  | X |  |  |  | 1.00% EO needles | Mirković et al., 2024 |
|  |  | Steam dist. |  | X |  |  |  | 0.80% EO cones | Mirković et al., 2024 |
|  | *eldarica* | Solvent batch |  | X |  |  |  | 14.0 % bark oil | Iravani & Zolfaghari, 2014 |
|  | *roxburghii* | Steam dist. |  | X |  |  |  | 0.60% EO needles | Baghat et al., 2018 |
|  | *occidentalis* | Steam dist. |  | X |  |  |  | 0.39% EO needles | Zanoni et al., 1990 |
| Germacrene D | *sylvestris* | Steam dist. |  | X |  |  |  | 3.80% EO needles | Mirković et al., 2024 |
|  |  | Steam dist. |  | X |  |  |  | 0.07% EO cones | Mirković et al., 2024 |
|  | *nigra* | Steam dist. |  | X |  |  |  | 16.34% EO needles | Mirković et al., 2024 |
|  |  | Steam dist. |  | X |  |  |  | 1.39% EO cones | Mirković et al., 2024 |
|  | *mugo* | Steam dist. |  | X |  |  |  | 5.50% EO needles | Mirković et al., 2024 |
|  |  | Steam dist. |  | X |  |  |  | 16.50% EO cones | Mirković et al., 2024 |
|  | *halepensis* | Steam dist. |  | X |  |  |  | 1.51% EO needles | Mirković et al., 2024 |
|  |  | Steam dist. |  | X |  |  |  | 0.20% EO cones | Mirković et al., 2024 |
|  | *occidentalis* | Steam dist. |  | X |  |  |  | 22.3% EO needles | Zanoni et al., 1990 |

Table (continued)

| **Compound** | **Pine sp** | **Extraction** | **Detected by** | | | | | **Content** | **References** |
| --- | --- | --- | --- | --- | --- | --- | --- | --- | --- |
|  |  |  | **GC/FID** | **GC/MS** | **HPLC/UV** | **HPLC/DAD** | **HPLC/MS** |  |  |
| α-Humulene | *sylvestris* | Steam dist. |  | X |  |  |  | 0.85% EO needles | Mirković et al., 2024 |
|  |  | Steam dist. |  | X |  |  |  | 1.56% EO cones | Mirković et al., 2024 |
|  | *nigra* | Steam dist. |  | X |  |  |  | 1.53% EO needles | Mirković et al., 2024 |
|  |  | Steam dist. |  | X |  |  |  | 2.33% EO cones | Mirković et al., 2024 |
|  | *mugo* | Steam dist. |  | X |  |  |  | 1.07% EO needles | Mirković et al., 2024 |
|  |  | Steam dist. |  | X |  |  |  | 3,70% EO cones | Mirković et al., 2024 |
|  | *halepensis* | Steam dist. |  | X |  |  |  | 3.85% EO needles | Mirković et al., 2024 |
|  |  | Steam dist. |  | X |  |  |  | 2.01% EO cones | Mirković et al., 2024 |
|  | *roxburghii* | Steam dist. |  | X |  |  |  | 3.1% EO needles | Sharma et al., 2020 |
|  |  | Steam dist. |  | X |  |  |  | 0.1% bark oil | Sharma et al., 2020 |
|  | *gerardiana* | Steam dist. |  | X |  |  |  | 0.4% EO needles | Sharma et al., 2020 |
| α-Muurolene | *sylvestris* | Steam dist. |  | X |  |  |  | 0.06% EO cones | Mirković et al., 2024 |
|  | *nigra* | Steam dist. |  | X |  |  |  | 0.04% EO needles | Mirković et al., 2024 |
|  |  | Steam dist. |  | X |  |  |  | 0.34% EO cones | Mirković et al., 2024 |
|  | *mugo* | Steam dist. |  | X |  |  |  | 3.47% EO needles | Mirković et al., 2024 |
|  |  | Steam dist. |  | X |  |  |  | 0.62% EO cones | Mirković et al., 2024 |
|  | *halepensis* | Steam dist. |  | X |  |  |  | 0.20% EO cones | Mirković et al., 2024 |
|  | *occidentalis* | Steam dist. |  | X |  |  |  | 0.30% EO needles | Zanoni et al., 1990 |
| δ-Cadinene | *sylvestris* | Steam dist. |  | X |  |  |  | 4.12% EO needles | Mirković et al., 2024 |
|  |  | Steam dist. |  | X |  |  |  | 0.05% EO cones | Mirković et al., 2024 |
|  | *nigra* | Steam dist. |  | X |  |  |  | 1.13% EO needles | Mirković et al., 2024 |
|  |  | Steam dist. |  | X |  |  |  | 0.36% EO cones | Mirković et al., 2024 |
| δ-Cadinene | *mugo* | Steam dist. |  | X |  |  |  | 3.70% EO needles | Mirković et al., 2024 |
|  |  | Steam dist. |  | X |  |  |  | 0.69% EO cones | Mirković et al., 2024 |
|  | *halepensis* | Steam dist. |  | X |  |  |  | 1.02% EO needles | Mirković et al., 2024 |
|  |  | Steam dist. |  | X |  |  |  | 0.45% EO cones | Mirković et al., 2024 |
|  | *occidentalis* | Steam dist. |  | X |  |  |  | 0.25% EO needles | Zanoni et al., 1990 |

Table (continued)

| **Compound** | | **Pine sp** | **Extraction** | **Detected by** | | | | | | **Content** | **References** |
| --- | --- | --- | --- | --- | --- | --- | --- | --- | --- | --- | --- |
|  |  |  |  | **GC/FID** | **GC/MS** | **HPLC/UV** | **HPLC/DAD** | **HPLC/MS** | |  |  |
| α-Cadinol | | *nigra* | Steam dist. |  | X |  |  |  | | 0.55% EO needles | Mirković et al., 2024 |
|  |  |  | Steam dist. |  | X |  |  |  | | 0.25% EO cones | Mirković et al., 2024 |
|  |  | *mugo* | Steam dist. |  | X |  |  |  | | 1.72% EO needles | Mirković et al., 2024 |
|  |  |  | Steam dist. |  | X |  |  |  | | 0.07% EO cones | Mirković et al., 2024 |
|  |  | *halepensis* | Steam dist. |  | X |  |  |  | | 0.21% EO needles | Mirković et al., 2024 |
|  |  |  | Steam dist. |  | X |  |  |  | | 0.09% EO cones | Mirković et al., 2024 |
|  |  | *roxburghii* | Steam dist. |  | X |  |  |  | | 2.7% EO needles | Sharma et al., 2020 |
|  |  |  | Steam dist. |  | X |  |  |  | | 0.2% bark oil | Sharma et al., 2020 |
|  |  | *wallichiana* | Steam dist. |  | X |  |  |  | | 2.2% EO needles | Sharma et al., 2020 |
|  |  |  | Steam dist. |  | X |  |  |  | | 3.5% bark oil | Sharma et al., 2020 |
|  |  | *gerardiana* | Steam dist. |  | X |  |  |  | | 0.5% EO needles | Sharma et al., 2020 |
| **Triterpenes** | | | | | | | | | | | |
| Betulin | | *pinaster* | Soxhlet |  | X |  |  |  | 425 μg/g bark^e^ | | Sousa et al, 2018 |
| **Polyphenols** | | | | | | | | | | | |
| Catechin | *pinaster* | | Soxhlet |  | X |  |  |  | NR^g^ | | Vieito et al, 2019 |
|  |  |  | Solvent batch |  |  | X |  |  | 4.1 % extract | | Iravani & Zolfaghari, 2014 |
|  |  |  | Solvent batch |  |  |  | X |  | 105-135 mg/L extract | | Ferreira Santos et al, 2020 |
|  |  |  | Solvent batch |  |  | X |  |  | 101-103 μg/g bark | | Gascon et al, 2018 |
|  |  |  | UAE/MAE^a^ |  |  | X |  |  | 3-5 mg/g bark | | Chupin et al, 2013 |
|  |  |  | Solvent batch |  |  |  |  | X | 2.3 μg/g bark | | Ramos et al, 2022 |
|  | *sylvestris* | | UAE/MAE^a^ |  |  |  | X |  |  | | Nisca et al., 2021 |
|  | *nigra* | | UAE/MAE^a^ |  |  |  | X |  | 0.60 bark^c^ | | Nisca et al., 2021 |
|  | *radiata* | | Solvent batch |  |  |  | X | X | 11.4-13.8 mg/g extract | | Bocalandro et al, 2012 |
|  | *densiflora* | | Solvent batch |  |  |  | X |  | 24.5 mg/g extract | | Kim et al, 2018 |
|  | *eldarica* | | Solvent batch |  |  | X |  |  | 3.4 % extract | | Iravani & Zolfaghari, 2014 |
|  | *pinea* | | Solvent batch |  |  | X |  |  | 98-106 μg/g bark | | Gascon et al, 2018 |
|  |  |  | Solvent batch |  |  |  |  | X | 2.8 μg/g bark | | Ramos et al, 2022 |
|  | *halepensis* | | Solvent batch |  |  | X |  |  | 40-52 μg/g bark | | Gascon et al, 2018 |
|  | *brutia* | | SFE^b^ |  |  | X |  |  | 3 mg/g extract | | Yesil-Celiktas et, 2009 |

Table (continued)

| **Compound** | | **Pine sp** | **Extraction** | | **Detected by** | | | | | | | | | | | | **Content** | | **References** |
| --- | --- | --- | --- | --- | --- | --- | --- | --- | --- | --- | --- | --- | --- | --- | --- | --- | --- | --- | --- |
|  |  |  |  |  | **GC/FID** | | **GC/MS** | | | **HPLC/UV** | | **HPLC/DAD** | | **HPLC/MS** | | |  |  |  |
| Epicatechin | *sylvestris* | | UAE/MAE^a^ | |  | |  | | |  | | X | |  | |  | | | Nisca et al., 2021 |
|  | *nigra* | | UAE/MAE^a^ | |  | |  | | |  | | X | |  | | 2.3-3.8 μg/g bark^e^ | | | Nisca et al., 2021 |
|  | *radiata* | | Solvent batch | |  | |  | | |  | | X | | X | | 2.8-3.3 mg/g extract | | | Bocalandro et al, 2012 |
|  | *pinaster* | | UAE/MAE^a^ | |  | |  | | | X | |  | |  | | 0.8-1.9 mg/g bark | | | Chupin et al, 2013 |
|  | *brutia* | | SFE^b^ | |  | |  | | | X | |  | |  | | 0.2 mg/g extract | | | Yesil-Celiktas et, 2009 |
| Quercetin | *pinaster* | | Soxhlet | |  | | X | | |  | |  | |  | | NR^g^ | | | Vieito et al, 2019 |
|  |  |  | Solvent batch | |  | |  | | |  | | X | |  | | 8.5-11.0 mg/L extract | | | Ferreira Santos et al, 2020 |
|  | *sylvestris* | | Solvent batch | |  | |  | | | X | |  | |  | | 4.0 %^f^ | | | Hamad et al, 2019 |
|  | *radiata* | | Solvent batch | |  | |  | | |  | | X | | X | | 3.7-4.3 mg/g extract | | | Bocalandro et al, 2012 |
| Epicatechin gallate | *pinaster* | | UAE/MAE^a^ | |  | |  | | | X | |  | |  | | 0.3-1.0 mg/g bark | | | Chupin et al, 2013 |
| Gallocatechin | *pinaster* | | Solvent batch | |  | |  | | |  | | X | |  | | 140-149 mg/L extract | | | Ferreira Santos et al, 2020 |
| Apigenin | *pinaster* | | Solvent batch | |  | |  | | |  | | X | |  | | 1.9-12.4 mg/L extract | | | Ferreira Santos et al, 2020 |
| Naringenin | *pinaster* | | Solvent batch | |  | |  | | |  | | X | |  | | 128-249 mg/L extract | | | Ferreira Santos et al, 2020 |
| Naringin | *sylvestris* | | Solvent batch | |  | |  | | | X | |  | |  | | 0.4 %^f^ | | | Hamad et al, 2019 |
| Taxifolin | *pinaster* | | | Soxhlet | |  | | X |  | |  | |  | | NR^g^ | | | Vieito et al, 2019 | |
|  |  |  |  | Solvent batch | |  | |  | X | |  | |  | | 6.7 % extract | | | Iravani & Zolfaghari, 2014 | |
|  |  |  |  | Solvent batch | |  | |  |  | | X | |  | | 73-464 mg/L extract | | | Ferreira Santos et al, 2020 | |
|  |  |  |  | Solvent batch | |  | |  | X | |  | |  | | 124-132 μg/g bark | | | Gascon et al, 2018 | |
|  |  |  |  | Solvent batch | |  | |  |  | |  | | X | | 1.5 μg/g bark | | | Ramos et al, 2022 | |
|  | *sylvestris* | | | Solvent batch | |  | |  | X | |  | |  | | 1.9 %^f^ | | | Hamad et al, 2019 | |
|  |  |  |  | ND^c^ | |  | |  |  | |  | | X | | NR^g^ | | | Saleem et al., 2003 | |
|  | *radiata* | | | Solvent batch | |  | |  |  | | X | | X | | 13.9-17.2 mg/g extract | | | Bocalandro et al, 2012 | |
|  | *densiflora* | | | Solvent batch | |  | |  |  | | X | |  | | 7.7 mg/g extract | | | Kim et al, 2018 | |
|  | *eldarica* | | | Solvent batch | |  | |  | X | |  | |  | | 2.1 % extract | | | Iravani & Zolfaghari, 2014 | |
|  | *pinea* | | | Solvent batch | |  | |  | X | |  | |  | | 105-119 μg/g bark | | | Gascon et al, 2018 | |
|  |  |  |  | Solvent batch | |  | |  |  | |  | | X | | 1.4 μg/g bark | | | Ramos et al, 2022 | |
|  | *halepensis* | | | Solvent batch | |  | |  | X | |  | |  | | 80-86 μg/g bark | | | Gascon et al, 2018 | |
|  | *brutia* | | | SFE^b^ | |  | |  | X | |  | |  | | 0.1 mg/g extract | | | Yesil-Celiktas et, 2009 | |
| Kaempferol | *sylvestris* | | | Solvent batch | |  | |  | X | |  | |  | | 0.8 %^f^ | | | Hamad et al, 2019 | |

Table (continued)

| **Compound** | | **Pine sp** | | **Extraction** | | **Detected by** | | | | | | | | | | | **Content** | | **References** |
| --- | --- | --- | --- | --- | --- | --- | --- | --- | --- | --- | --- | --- | --- | --- | --- | --- | --- | --- | --- |
|  |  |  |  |  |  | **GC/FID** | | **GC/MS** | | | **HPLC/UV** | | **HPLC/DAD** | | **HPLC/MS** | |  |  |  |
| Resveratrol | *pinaster* | | | | Solvent batch | |  | |  |  | | X | |  | | 3.8-18.9 mg/L extract | | Ferreira Santos et al, 2020 | |
| Myricetin | *sylvestris* | | | | Solvent batch | |  | |  | X | |  | |  | | 20.8 %^f^ | | Hamad et al, 2019 | |
| Eleutheroside | *sylvestris* | | | | Solvent batch | |  | |  | X | |  | |  | | 9.4 %^f^ | | Hamad et al, 2019 | |
| Butein | *sylvestris* | | | | Solvent batch | |  | |  | X | |  | |  | | 1.1 %^f^ | | Hamad et al, 2019 | |
| Luteolin | *sylvestris* | | | | Solvent batch | |  | |  | X | |  | |  | | 0.1 %^f^ | | Hamad et al, 2019 | |
| Vanillin | *densiflora* | | | | Solvent batch | |  | |  |  | | X | |  | | 2.3 mg/g extract | | Kim et al, 2018 | |
| Rutin | ND^c^ | | | | ND^c^ | |  | |  |  | |  | | X | | NR^g^ | | Cadiz-Gurrea et al, 2014 | |
| **Procyanidins (Types)** | | | | | | | | | | | | | | | | | | | |
| A1-A8 | ND^c^ | | | | ND^c^ | |  | |  |  | |  | | X | | NR^g^ | | Cadiz-Gurrea et al, 2014 | |
| A1 Dimer | *pinaster* | | | | Solvent batch | |  | |  |  | |  | | X | | 0.9 μg/g bark | | Ramos et al, 2022 | |
|  | *pinea* | | | | Solvent batch | |  | |  |  | |  | | X | | 1.2 μg/g bark | | Ramos et al, 2022 | |
| A2 | *halepensis* | | | | Solvent batch | |  | |  | X | |  | |  | | 21-23 μg/g bark | | Gascon et al, 2018 | |
| B1-B2 | ND^c^ | | | | ND^c^ | |  | |  |  | |  | | X | | NR^g^ | | Cadiz-Gurrea et al, 2014 | |
| B1 Dimer | | | *pinaster* | | Solvent batch | |  | |  | X | |  | |  | | 5-6 μg/g bark | | Gascon et al, 2018 | |
|  |  |  |  |  | Solvent batch | |  | |  |  | |  | | X | | 0.4 μg/g bark | | Ramos et al, 2022 | |
|  |  |  | *pinea* | | Solvent batch | |  | |  | X | |  | |  | | 12-16 μg/g bark | | Gascon et al, 2018 | |
|  |  |  |  |  | Solvent batch | |  | |  |  | |  | | X | | 0.5 μg/g bark | | Ramos et al, 2022 | |
|  |  |  | *halepensis* | | Solvent batch | |  | |  | X | |  | |  | | 7 μg/g bark | | Gascon et al, 2018 | |
| B2 Dimer | | | *pinaster* | | Solvent batch | |  | |  | X | |  | |  | | 41-43 μg/g bark | | Gascon et al, 2018 | |
|  |  |  |  |  | Solvent batch | |  | |  |  | |  | | X | | 1.4 μg/g bark | | Ramos et al, 2022 | |
|  |  |  | *pinea* | | Solvent batch | |  | |  | X | |  | |  | | 28-29 μg/g bark | | Gascon et al, 2018 | |
|  |  |  |  |  | Solvent batch | |  | |  |  | |  | | X | | 1.6 μg/g bark | | Ramos et al, 2022 | |
|  |  |  | *halepensis* | | Solvent batch | |  | |  | X | |  | |  | | 11 μg/g bark | | Gascon et al, 2018 | |
| B2 Trimer | | | *pinaster* | | Solvent batch | |  | |  |  | |  | | X | | 0.8 μg/g bark | | Ramos et al, 2022 | |
|  |  |  | *pinea* | | Solvent batch | |  | |  |  | |  | | X | | 0.6 μg/g bark | | Ramos et al, 2022 | |
| B3 Trimer | | | *pinaster* | | Solvent batch | |  | |  |  | |  | | X | | 0.9 μg/g bark | | Ramos et al, 2022 | |
|  |  |  | *pinea* | | Solvent batch | |  | |  |  | |  | | X | | 1.3 μg/g bark | | Ramos et al, 2022 | |
| B4 Dimer | | | *pinaster* | | Solvent batch | |  | |  |  | |  | | X | | 0.2 μg/g bark | | Ramos et al, 2022 | |
|  |  |  | *pinea* | | Solvent batch | |  | |  |  | |  | | X | | 0.1 μg/g bark | | Ramos et al, 2022 | |

Table (continued)

| **Compound** | **Pine sp** | | **Extraction** | | **Detected by** | | | | | | | | | | | **Content** | | **References** |
| --- | --- | --- | --- | --- | --- | --- | --- | --- | --- | --- | --- | --- | --- | --- | --- | --- | --- | --- |
|  |  |  |  |  | **GC/FID** | | **GC/MS** | | | **HPLC/UV** | | **HPLC/DAD** | | **HPLC/MS** | |  |  |  |
| B4 Trimer | | *pinaster* | | Solvent batch | |  | |  |  | |  | | X | | 0.2 μg/g bark | | Ramos et al, 2022 | |
|  |  | *pinea* | | Solvent batch | |  | |  |  | |  | | X | | 0.8 μg/g bark | | Ramos et al, 2022 | |
| C | | ND^c^ | | ND^c^ | |  | |  |  | |  | | X | | NR^g^ | | Cadiz-Gurrea et al, 2014 | |
| Trimers | | ND^c^ | | ND^c^ | |  | |  |  | |  | | X | | NR^g^ | | Cadiz-Gurrea et al, 2014 | |
| Flavan-3-ols (1-6) | | ND^c^ | | ND^c^ | |  | |  |  | |  | | X | | NR^g^ | | Cadiz-Gurrea et al, 2014 | |
| Epicatechin oligomers (1-9) | | ND^c^ | | ND^c^ | |  | |  |  | |  | | X | | NR^g^ | | Cadiz-Gurrea et al, 2014 | |
| Caffeic | | *pinaster* | | Soxhlet | |  | | X |  | |  | |  | | 7 μg/g bark^e^ | | Sousa et al, 2018 | |
|  |  |  |  | Solvent batch | |  | |  | X | |  | |  | | 1,9 % extract | | Iravani & Zolfaghari, 2014 | |
|  |  |  |  | Solvent batch | |  | |  |  | | X | |  | | 4.2-13.8 mg/L extract | | Ferreira Santos et al, 2020 | |
|  |  | *eldarica* | | Solvent batch | |  | |  | X | |  | |  | | 1.2 % extract | | Iravani & Zolfaghari, 2014 | |
| Ferulic | | *pinaster* | | Soxhlet | |  | |  | X | |  | |  | | NR^g^ | | Vieito et al, 2019 | |
|  |  |  |  | Soxhlet | |  | | X |  | |  | |  | | 58 μg/g bark^e^ | | Sousa et al, 2018 | |
|  |  |  |  | Solvent batch | |  | |  | X | |  | |  | | 2.3 % extract | | Iravani & Zolfaghari, 2014 | |
|  |  |  |  | Solvent batch | |  | |  |  | | X | |  | | 9.7-24.5 mg/L extract | | Ferreira Santos et al, 2020 | |
|  |  | *eldarica* | | Solvent batch | |  | |  | X | |  | |  | | 2.3 % extract | | Iravani & Zolfaghari, 2014 | |
|  |  | *pinea* | | Soxhlet | |  | | X |  | |  | |  | | 29 μg/g bark^e^ | | Sousa et al, 2018 | |
| Protocatechuic | | *densiflora* | | Solvent batch | |  | |  |  | | X | |  | | 7.7 mg/g extract | | Kim et al, 2018 | |
|  |  | *pinaster* | | Solvent batch | |  | |  |  | |  | | X | | 0.35 μg/g bark | | Ramos et al, 2022 | |
|  |  | *pinea* | | Solvent batch | |  | |  |  | |  | | X | | 0.40 μg/g bark | | Ramos et al, 2022 | |
|  |  | *sylvestris* | | ND^c^ | |  | |  |  | |  | | X | | NR | | Saleem et al, 2002 | |
| Chlorogenic | | *pinaster* | | Solvent batch | |  | |  |  | | X | |  | | 5.8-17.2 mg/L extract | | Ferreira Santos et al, 2020 | |
| Ellagic | | *pinaster* | | Solvent batch | |  | |  |  | | X | |  | | 53.6-124.4 mg/L extract | | Ferreira Santos et al, 2020 | |
| 3,4-Dihydrobenzoic | | *radiata* | | Solvent batch | |  | |  |  | | X | | X | | 0.9-1.3 mg/g extract | | Bocalandro et al, 2012 | |
|  |  | *pinaster* | | Solvent batch | |  | |  |  | | X | |  | | 29.7-64.1 % extract | | Ferreira Santos et al, 2020 | |
| p-Hydroxybenzoic | | *radiata* | | Solvent batch | |  | |  |  | | X | | X | | 7.6-8.6 mg/g extract | | Bocalandro et al, 2012 | |
| Gallic | | *pinaster* | | Soxhlet | |  | |  | X | |  | |  | | NR^g^ | | Vieito et al, 2019 | |
| Rosmarinic | | *pinaster* | | Solvent batch | |  | |  |  | | X | |  | |  | | Ferreira Santos et al, 2020 | |
| Syringic | | *pinaster* | | Soxhlet | |  | | X |  | |  | |  | | 1 μg/g bark^e^ | | Sousa et al, 2018 | |
|  |  | *radiata* | | Solvent batch | |  | |  |  | | X | | X | | 0.8-1.1 mg/g extract | | Bocalandro et al, 2012 | |

Table (continued)

| **Compound** | **Pine sp** | | **Extraction** | | **Detected by** | | | | | | | | | | | **Content** | | **References** |
| --- | --- | --- | --- | --- | --- | --- | --- | --- | --- | --- | --- | --- | --- | --- | --- | --- | --- | --- |
|  |  |  |  |  | **GC/FID** | | **GC/MS** | | | **HPLC/UV** | | **HPLC/DAD** | | **HPLC/MS** | |  |  |  |
| Vanillic | | *pinaster* | | Soxhlet | |  | | X |  | |  | |  | | 6 μg/g bark^e^ | | Sousa et al, 2018 | |
|  |  |  |  | Solvent batch | |  | |  |  | | X | |  | | 3.0-10.5 mg/L extract | | Ferreira Santos et al, 2020 | |
| Quinic | | *pinaster* | | Solvent batch | |  | |  |  | |  | | X | | 6 μg/g bark | | Ramos et al, 2022 | |
|  |  | *pinea* | | Solvent batch | |  | |  |  | |  | | X | | 8 μg/g bark | | Ramos et al, 2022 | |
| Cinnamic | | *pinaster* | | Solvent batch | |  | |  |  | | X | |  | | 5.4-53.4 mg/L extract | | Ferreira Santos et al, 2020 | |
| Homovanillic | | *radiata* | | Solvent batch | |  | |  |  | | X | | X | | 5.3-6.7 mg/g extract | | Bocalandro et al, 2012 | |
| Kaurenoic | | *sylvestris* | | Solvent batch | |  | |  | X | |  | |  | | 3.2 %^d^ | | Hamad et al, 2019 | |
| p-Coumaric | | *pinaster* | | Soxhlet | |  | | X |  | |  | |  | | 8 μg/g bark^e^ | | Sousa et al, 2018 | |
| **Long-chain alcohols** | | | | | | | | | | | | | | | | | | |
| 1-Hexadecanol | | *pinaster* | | Soxhlet | |  | | X |  | |  | |  | | 11 μg/g bark^e^ | | Sousa et al, 2018 | |
|  |  | *pinea* | | Soxhlet | |  | | X |  | |  | |  | | 8 μg/g bark^e^ | | Sousa et al, 2018 | |
| Octadec-9-en-1-ol | | *pinaster* | | Soxhlet | |  | | X |  | |  | |  | | 11 μg/g bark^e^ | | Sousa et al, 2018 | |
|  |  | *pinea* | | Soxhlet | |  | | X |  | |  | |  | | 8 μg/g bark^e^ | | Sousa et al, 2018 | |
| 1-Octadecanol | | *pinaster* | | Soxhlet | |  | | X |  | |  | |  | | 6 μg/g bark^e^ | | Sousa et al, 2018 | |
|  |  | *pinea* | | Soxhlet | |  | | X |  | |  | |  | | 8 μg/g bark^e^ | | Sousa et al, 2018 | |
| 1-Docosanol | | *pinaster* | | Soxhlet | |  | | X |  | |  | |  | | 34 μg/g bark^e^ | | Sousa et al, 2018 | |
|  |  | *pinea* | | Soxhlet | |  | | X |  | |  | |  | | 71 μg/g bark^e^ | | Sousa et al, 2018 | |
| 1-Tetracosanol | | *pinaster* | | Soxhlet | |  | | X |  | |  | |  | | 62 μg/g^c^ bark^e^ | | Sousa et al, 2018 | |
|  |  | *pinea* | | Soxhlet | |  | | X |  | |  | |  | | 124 μg/g bark^e^ | | Sousa et al, 2018 | |
| **Sterols** | | | | | | | | | | | | | | | | | | |
| Campesterol | | *pinaster* | | Soxhlet | |  | | X |  | |  | |  | | 33 μg/g bark^e^ | | Sousa et al, 2018 | |
|  |  | *pinea* | | Soxhlet | |  | | X |  | |  | |  | | 18 μg/g bark^e^ | | Sousa et al, 2018 | |
| β-Sitosterol | | *pinaster* | | Soxhlet | |  | | X |  | |  | |  | | 434 μg/g bark^e^ | | Sousa et al, 2018 | |
|  |  | *pinea* | | Soxhlet | |  | | X |  | |  | |  | | 224 μg/g bark^e^ | | Sousa et al, 2018 | |
| Stigmast-4-en-3-one | | *pinaster* | | Soxhlet | |  | | X |  | |  | |  | | 120 μg/g bark^e^ | | Sousa et al, 2018 | |

**Legend:** a. UAE/MAE: Ultrasound Assisted/Microwave Assisted; b. SFE: Supercritical Fluid Extraction; c. ND: Not described; d. n-hexane extract; e. dichloromethane extract; f. ethanol: water (70:30) extract; g. NR = Not Reported. h. TCD detector was used. EO = Essential oil.
